# Peri‐operative tobacco cessation interventions: a systematic review and meta‐analysis

**DOI:** 10.1111/anae.16120

**Published:** 2023-09-01

**Authors:** S. Harrogate, J. Barnes, K. Thomas, A. Isted, G. Kunst, S. Gupta, S. Rudd, T. Banerjee, R. Hinchliffe, R. Mouton

**Affiliations:** ^1^ Elizabeth Blackwell Institute University of Bristol Bristol UK; ^2^ Department of Anaesthesia, North Bristol NHS Trust Bristol UK; ^3^ Department of Population Health Sciences, Bristol Medical School University of Bristol Bristol UK; ^4^ Department of Anaesthesia, King's College Hospital NHS Foundation Trust London UK; ^5^ School of Cardiovascular and Metabolic Medicine and Sciences, King's College London London UK; ^6^ Department of Anaesthesia, King's College Hospital NHS Foundation Trust London UK; ^7^ Department of Anaesthesia University Hospitals Bristol and Weston NHS Foundation Trust Bristol UK; ^8^ North Bristol NHS Trust Bristol UK; ^9^ University of Leicester Leicester UK; ^10^ Department of Translational Health Sciences, Bristol Medical School University of Bristol Bristol UK; ^11^ Department of Vascular Services, North Bristol NHS Trust Bristol UK; ^12^ Department of Translational Health Sciences, Bristol Medical School University of Bristol Bristol UK; ^13^ Department of Anaesthesia, North Bristol NHS Trust Bristol UK

**Keywords:** abstinence, addiction, meta‐analysis, peri‐operative tobacco cessation interventions, systematic review

## Abstract

Tobacco smoking is associated with a substantially increased risk of postoperative complications. The peri‐operative period offers a unique opportunity to support patients to stop tobacco smoking, avoid complications and improve long‐term health. This systematic review provides an up‐to‐date summary of the evidence for tobacco cessation interventions in surgical patients. We conducted a systematic search of randomised controlled trials of tobacco cessation interventions in the peri‐operative period. Quantitative synthesis of the abstinence outcomes data was by random‐effects meta‐analysis. The primary outcome of the meta‐analysis was abstinence at the time of surgery, and the secondary outcome was abstinence at 12 months. Thirty‐eight studies are included in the review (7310 randomised participants) and 26 studies are included in the meta‐analysis (5969 randomised participants). Studies were pooled for subgroup analysis in two ways: by the timing of intervention delivery within the peri‐operative period and by the intensity of the intervention protocol. We judged the quality of evidence as moderate, reflecting the degree of heterogeneity and the high risk of bias. Overall, peri‐operative tobacco cessation interventions increased successful abstinence both at the time of surgery, risk ratio (95%CI) 1.48 (1.20–1.83), number needed to treat 7; and 12 months after surgery, risk ratio (95%CI) 1.62 (1.29–2.03), number needed to treat 9. More work is needed to inform the design and optimal delivery of interventions that are acceptable to patients and that can be incorporated into contemporary elective and urgent surgical pathways. Future trials should use standardised outcome measures.

## Introduction

Tobacco smoking is the single biggest cause of preventable illness worldwide [1]. The decline in overall smoking prevalence is steady but slow; despite active public health campaigns, 13.3% of adults in England continue to smoke tobacco [2]. It remains especially common in populations that are poorly served by existing healthcare interventions; this widens health inequalities [3]. Tobacco smoking increases the likelihood that patients will suffer cardiovascular and pulmonary complications, wound infections and intensive care admissions after surgery [4, 5]. The prevalence of smoking is very high in some specific surgical populations; for example, a third of people undergoing vascular surgery in the UK and up to 50% of trauma patients in the USA are current smokers [6, 7]. With over 300 million operations performed annually worldwide, peri‐operative smoking presents a clear problem which, if addressed, has the potential to reduce demand on resource‐limited systems and improve healthcare outcomes for a large number of people [8].

The peri‐operative period is an important opportunity to approach patients about quitting tobacco. Patients referred for surgery experience a shift in focus towards their immediate health and well‐being, leading to increased motivation and more favourable attitudes towards improving their health behaviours, including smoking cessation [9]. Nevertheless, current peri‐operative smoking cessation support services are underused: only around 5% of surgical patients who are referred to a stop‐smoking service take up the referral [10, 11].

Recently, there has been an important shift in the approach to reducing harm from tobacco smoking. The latest guideline from the National Institute for Health and Care Excellence (NICE) advises a treatment strategy that aims to treat tobacco addiction, rather than nicotine use, through prescriptions of licensed nicotine‐containing products [12]. In contemporary practice, nicotine‐containing electronic cigarettes (e‐cigarettes) appear to be one of the most effective pharmacotherapies for smoking cessation. A 2021 Cochrane systematic review concluded that the use of e‐cigarettes increases abstinence from tobacco smoking when compared with other forms of nicotine replacement therapy in the general population [13]. The Office for Health Improvement and Disparities has concluded that e‐cigarettes pose only a small fraction of the risks of smoking [14]. E‐cigarettes are, therefore, a new, effective and potentially transformative smoking cessation intervention.

The last Cochrane review of pre‐operative smoking cessation interventions was published in 2014 [15]. The authors found some evidence that pre‐operative behavioural support and nicotine replacement therapy increased short‐term smoking cessation and might reduce postoperative morbidity. Since then, many further randomised controlled trials have been published addressing this question [6, 16, 17, 18, 19, 20, 21, 22, 23], including trials examining the effectiveness of e‐cigarettes, during the peri‐operative period [24]. Furthermore, the 2014 review focused only on pre‐operative interventions, excluding studies with tobacco cessation protocols that start or continue during and after surgery [15]. In practice, quality standards or clinical urgency demand short time frames between referral and surgery. In these circumstances, there is little time to implement a purely pre‐operative smoking cessation intervention. It is also worth noting that the pre‐operative period is only one part of the overall peri‐operative care.

The aim of our review was to assess the impact of all peri‐operative tobacco cessation interventions on abstinence from tobacco smoking at the time of surgery and 12 months after.

## Methods

We undertook a systematic literature search using standard methods [25] and report the work according to the preferred reporting items for systematic reviews and meta‐analyses (PRISMA) statements [26].

We searched: EMBASE (Ovid); Medline (Ovid); Pubmed; CINAHL (Ebsco); PsychInfo (ProQuest); the Cochrane CENTRAL Register of Controlled Trials; and the ISRCTN registry, from inception to 31 August 2022. The search strategy (online Supporting Information Figure S1), developed in collaboration with an experienced medical librarian, is reported in line with the PRISMA‐S protocol [27]. The search included text words and subject headings for ‘smoking’, ‘cessation’ and ‘peri‐operative’, which were translated across all databases. Search results were limited to English language and humans.

We included randomised controlled trials, with no restrictions on the country of study or the type of healthcare setting; adult patients (aged ≥18 y) who were current smokers, undergoing surgery (in any speciality, emergency and/or elective). We included any tobacco cessation intervention, used alone or in combination, delivered during the peri‐operative period, with ‘usual care’ as the comparator. The outcome of the systematic review was peri‐operative smoking cessation. The primary outcome of the meta‐analysis was abstinence at the time of surgery, and the secondary outcome was abstinence at 12 months.

The following definitions were applied: ‘peri‐operative’ was defined as the time‐period “from the moment of contemplation of surgery until full recovery” [28]. ‘Smoking’ or ‘tobacco smoking’, was defined as inhalational tobacco use (including smoking cigarettes, cigars, cigarillos, pipe tobacco and other forms of inhalational tobacco as described by the World Health Organization) [29]. ‘Nicotine replacement therapy’, ‘abstinence’, ‘point prevalence abstinence’ and ‘prolonged abstinence’ were defined according to the Cochrane Tobacco Addiction Group [30]. We included abstinence assessed by any method, including self‐reporting and biochemical verification.

EndNote [31] was used for reference management and de‐duplication. The retrieved references were imported into Rayyan [32] for subsequent screening. Titles and abstracts of all identified citations were divided and screened for eligibility by five reviewers (SH, JB, AI, SG and TB). Each abstract was cross‐checked by a second independent, blinded reviewer. Full texts of the selected studies were then independently screened by two blinded reviewers. The references and onward citations of all papers included for full‐text review were identified using the citationchaser tool [33] and screened by SH. Excluded studies are listed in online Supporting Information Table S1. Three reviewers extracted data (SH, JB, AI). A standardised data extraction form was used (online Supporting Information Appendix S1) and data were cross‐checked by a second reviewer. Authors were contacted directly when information was missing or unclear. Disagreements were resolved by group consensus. Data were recorded in Microsoft Excel™ [34].

Some studies examined multimodal treatment programmes, for example, cardiac rehabilitation or enhanced recovery protocols which included a peri‐operative smoking cessation intervention. Such studies were included if smoking cessation was a prespecified outcome. Other studies examined smoking cessation interventions in mixed cohorts that comprised a combination of participants who were managed surgically and non‐surgically, or participants who did and did not smoke. Such studies were included if they met one or more of the following criteria: smoking patients were a prespecified subgroup; smoking cessation was a prespecified outcome; over 80% of patients in the cohort met the relevant smoking or surgical inclusion criteria. Studies that met the criteria above are listed in online Supporting Information Table S2.

We conducted a descriptive analysis of the study methodology, intervention programmes and outcome measures. Additionally, we performed a meta‐analysis of abstinence at the time of surgery and at 12 months postoperatively. The prevalence of tobacco cessation was summarised using risk ratios (95%CI) as recommended by the Cochrane Tobacco Addiction Group [30]. Where appropriate, study data were pooled, using Mantel–Haenszel random effects meta‐analysis using RevMan 5 [35]. An available‐case analysis was used wherein the following participants were excluded from the denominator: those for whom surgery was cancelled or postponed; those who died before follow‐up; and those who were either mistakenly included or withdrew before receiving the intervention [25]. All other participants lost to follow‐up were assumed to be smoking, as is common practice in trials of smoking cessation interventions [15, 36]. Where studies assigned patients to multiple intervention groups, we pooled the results from all intervention groups and compared them to the control group for the purpose of meta‐analysis, as recommended by Higgins [25].

We conducted subgroup analyses in which studies were pooled according to the timeline of intervention delivery within the peri‐operative period (pre‐operative only, intra‐operative only, postoperative only, or both pre‐ and postoperative) and by the intensity of the intervention protocol (intensive smoking cessation intervention, short intervention, or other) [37].

We performed sensitivity analyses excluding pilot studies, and studies from mixed cohorts that included only a subgroup of smokers. To assess the impact of missing data, we also performed a sensitivity analysis excluding trials with more than 20% drop‐out. Heterogeneity was assessed using the chi^2^ statistic. We used the Cochrane Risk of Bias 2 tool [38] to assess risk of bias and produced funnel plots for each outcome to look for evidence of reporting bias.

## Results

**Figure 1:**
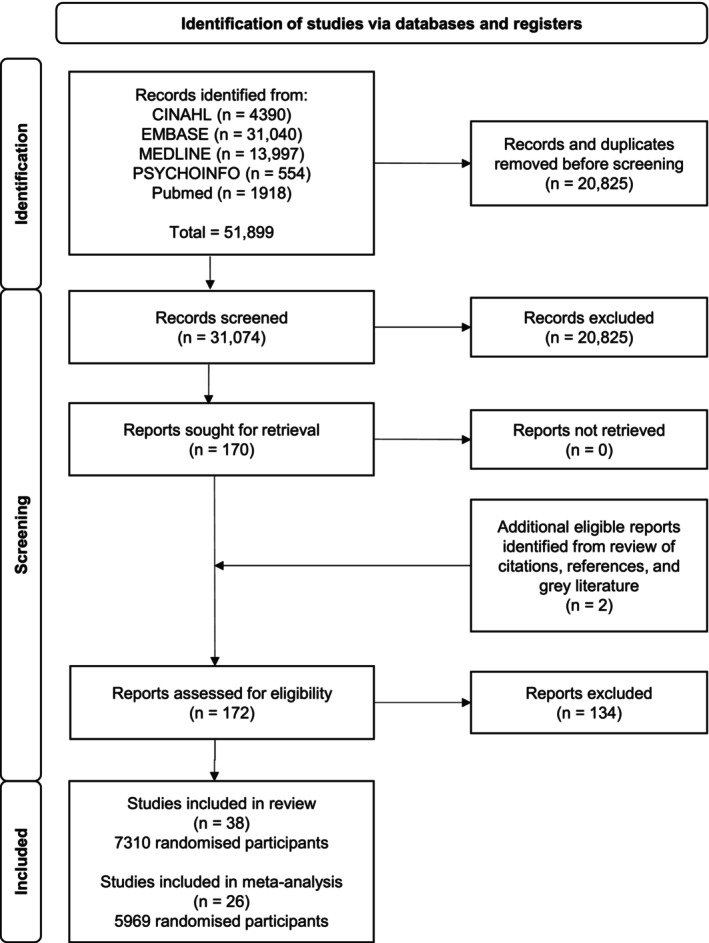
Study flow diagram.

The initial search strategy produced 51,899 citations and the screening process is reported in detail in Figure 1. We identified 170 citations for full‐text review. Thirty‐eight studies are included in the systematic review (Tables 1 and 2) [6, 16, 17, 20, 21, 22, 23, 39, 40, 41, 42, 43, 44, 45, 46, 47, 48, 49, 50, 51, 52, 53, 54, 55, 56, 57, 58, 59, 60, 61, 62, 63, 64, 65, 66, 67, 68]. Detailed descriptions of the interventions and outcomes are in online Supporting Information Table S3. Seven studies had > 20% loss to follow‐up, while 23 studies randomised < 80% of eligible participants (online Supporting Information Table S4). Twenty‐six studies are included in the meta‐analysis [16, 17, 20, 21, 23, 24, 39, 40, 45, 46, 47, 48, 51, 53, 54, 55, 56, 57, 58, 60, 61, 62, 63, 65, 67, 68]; of these, 21 examined the primary outcome while 11 examined the secondary outcome.

**Table 1:** Characteristics of the 38 randomised controlled trials included in the systematic review: population, intervention, control, and primary outcomes.

Intervention time‐point	Study	Intervention summary	Control treatment	Primary outcome
Pre‐operative only	McHugh et al. [47]	Multicomponent In‐person counselling programme Other: record cards	Usual care	Cardiac risk factors (including smoking abstinence) at time of surgery
	Myles et al. [51]	PT: bupropion Brief advice Written information Telephone counselling session	Placebo Brief advice Written information Telephone counselling session	Daily cigarette consumption at hospital admission
	Andrews et al. [40]	Other: letter from consultant surgeon SSS information	Usual care	Abstinence at time of surgery
	Sorensen et al. [58]	Two groups: Brief advice plus: Group 1: telephone counselling session Group 2: in‐person counselling programme, NRT	Usual care	Abstinence at time of surgery
	Thomsen et al. [60]	In‐person counselling session NRT	Usual care	Peri‐operative complications
	Warner et al. [61]	NRT: nicotine lozenge	Placebo: lozenge	Abstinence at time of surgery
	Shi et al. [56]	Brief advice Other: carbon monoxide testing advice	Brief advice	Carbon monoxide levels on the morning of surgery
	Warner et al. [63]	Other: decision aid in pre‐operative assessment clinic	Usual care	Decisional quality and patient involvement in decision making
	Bohlin et al. [21]	Three groups: Group 1: written information Group 2: other – doctor informed of smoking status Group 3: written information and other – doctor informed	Usual care	Abstinence at multiple peri‐operative time points
	Webb et al. [65]	Written information NRT	Usual care	Abstinence at time of surgery
	Rojewski et al. [55]	Other: monetary payment delivered contingent on abstinence In‐person counselling schedule NRT	In‐person counselling schedule NRT	Abstinence at time of surgery
Pre‐ and postoperative	Møller et al. [48]	In‐person counselling schedule NRT	Usual care	Peri‐operative complications
	Ratner et al. [53]	Brief advice SSS information Written information In‐person and telephone counselling schedule	Usual care	Abstinence at time of surgery
	Wolfenden et al. [67]	Non‐dependent smokers: Digital counselling session Written material Prompt for brief advice Telephone counselling programme Dependent smokers: As above plus NRT	Usual care	Abstinence at time of surgery
	Lindström et al. [46]	In‐person and/or telephone counselling schedule SSS information NRT	Usual care	Peri‐operative complications
	Warner et al. [62]	Brief advice SSS information and optional referral Written information SSS referral	Brief advice	Quitline use
	Wong et al. [68]	In‐person counselling schedule PT: varenicline	In‐person counselling schedule Placebo	Abstinence 12 months postoperatively
	Lee et al. [45]	Brief advice Written information SSS referral NRT	Usual care	Abstinence at time of surgery
	Ostroff et al. [16]	Brief advice NRT Digital counselling programme Other: digital scheduled reduced smoking programme	Brief advice NRT Digital counselling programme	Abstinence at time of surgery, 3 and 6 months postoperatively
	Kehlet et al. [43]	In‐person counselling schedule NRT	Usual care NRT	Peri‐operative complications
	Wong et al. [20]	In‐person counselling session PT: varenicline Written material SSS Referral	Brief advice SSS information	Abstinence at 12 months postoperatively
	Lee et al. [24]	Two groups: Brief advice Written information SSS referral Plus: Group 1: NRT: nicotine patch Group 2: NRT: e‐cigarettes tapering dose	Brief advice Written information SSS referral	Abstinence at time of surgery
	Lauridsen et al. [22]	Multicomponent: In‐person counselling programme NRT	Usual care	Peri‐operative complications
	Webb et al. [23]	Written information NRT SSS information Telephone counselling session	Usual care	Abstinence at time of surgery
Intra‐operative only	Myles [49]	Audiotape message during general anaesthesia	Sham intervention	Desire to quit smoking 1 day postoperatively
	Hughes et al. [42]	Audiotape message during general anaesthesia	Sham intervention	Abstinence 1 month postoperatively
	Myles et al. [50]	Audiotape message during general anaesthesia	Sham intervention	Abstinence at 2 and 6 months postoperatively
Postoperative only	Rigotti et al. [54]	Digital, in‐person and telephone counselling programme	Usual care	Abstinence at 12 months and 5.5 y postoperatively
	Stanislaw et al. [59]	In‐person and telephone counselling programme Written information Other: relaxation audiotape	Usual care	Abstinence at first post‐discharge visit
	Wewers et al. [66]	In‐person and telephone counselling programme Written information Other: relaxation audiotape	Usual care	Abstinence at first post‐discharge visit
	Allen et al. [39]	Multicomponent: In‐person and telephone counselling programme Written information	Usual care	Cardiac risk factors (including smoking abstinence) at 12 months postoperatively
	Simon et al. [57]	In‐person, digital and telephone counselling programme NRT Other: psychology referral if required Written information	Brief advice Written information	Abstinence at 6 and 12 months
	Griebel et al. [41]	In person and telephone counselling programme Written information	Usual care	Smoking status (7‐day abstinence) at outpatient visit 6 weeks after discharge Change in number of cigarettes smoked per day, documented smoking cessation intervention on patient chart
	Warner et al. [64]	NRT: nicotine patches	Placebo: patches	Perceived stress score
	Nåsell et al. [52]	In‐person and telephone counselling programme NRT	Usual care	Prevalence of one or more post operative complication at 2 and 6 weeks postoperatively Abstinence from smoking at 2 and 6 weeks postoperatively
	Kadda et al. [17]	Multicomponent: In‐person and telephone counselling programme	Usual care	Incidence of non‐fatal cardiovascular events at 1 year postoperatively
	Krebs et al. [44]	Telephone counselling programme PT/NRT: varenicline or bupropion and/or NRT Other: digital smoking cues coping skills game	Telephone counselling programme PT/NRT: varenicline or bupropion and/or NRT	Feasibility and game‐use metrics
	Matuszewski et al. [6]	Two intervention groups: Group 1: Brief advice SSS referral Group 2: Brief advice SSS referral In‐person and telephone counselling programme	Usual care	Abstinence at 3 and 6 months postoperatively

‘Multicomponent’ denotes an intervention programme consisting of multiple different interventions, e.g. addressing several cardiac risk factors, or smoking and alcohol. For such interventions, only the smoking cessation components are concisely summarised in this table. ‘Abstinence’ is abstinence from tobacco smoking. ‘Programme’ denotes two or more scheduled events. ‘Session’ denotes a single event. ‘Digital’ includes video, computer programmes or application on mobile device. PT, pharmacotherapy (varenicline, bupropion); SSS, stop smoking service, including quitline; NRT, nicotine replacement therapy offered or provided on a generic or personalised schedule.

**Table 2:** Characteristics of the 38 randomised controlled trials included in the systematic review: publication and participant details.

Intervention time‐point	Study	Year and country of publication	Population: type of surgery	Number of randomised trial participants	Number of smokers
Pre‐operative only	McHugh et al. [47]	2001 UK	Cardiac (CABG only)	98	23
	Myles et al. [51]	2004 Australia	Elective surgery, mixed	47	
	Andrews et al. [40]	2006 UK	Elective surgery, mixed	102	
	Sorensen et al. [58]	2007 Denmark	Elective general surgery	213	
	Thomsen et al. [60]	2010 Denmark	Breast surgery (cancer and other)	130	
	Warner et al. [61]	2012 USA	Elective surgery, mixed	46	
	Shi et al. [56]	2013 USA	Elective surgery, mixed	169	
	Warner et al. [63]	2015 USA	Elective surgery, mixed	121	
	Bohlin et al. [21]	2020 Sweden	Gynaecological surgery (cancer and other)	651	
	Webb et al. [65]	2020 Australia	Elective surgery, mixed	600	
	Rojewski et al. [55]	2021 USA	Cancer surgery, mixed	40	
Pre‐ and postoperative	Møller et al. [48]	2002 Denmark	Elective orthopaedic	120	
	Ratner et al. [53]	2004 Canada	Elective surgery, mixed (including cardiac)	237	
	Wolfenden et al. [67]	2005 Australia	Elective surgery, mixed (not cardiac)	210	
	Lindström et al. [46]	2008 Sweden	Elective orthopaedic and general surgery	117	
	Warner et al. [62]	2011 USA	Elective surgery, mixed	300	
	Wong et al. [68]	2012 Canada	Elective surgery, mixed (not cardiac)	286	
	Lee et al. [45]	2013 Canada	Elective surgery, mixed	168	
	Ostroff et al. [16]	2014 USA	Cancer surgery, mixed	185	
	Kehlet et al. [43]	2015 Denmark	Vascular surgery	32	
	Wong et al. [20]	2017 Canada	Elective surgery, mixed (not cardiac)	296	
	Lee et al. [24]	2018 USA	Elective surgery, mixed	30	
	Lauridsen et al. [22]	2022 Denmark	Radical cystectomy for bladder cancer	104	82
	Webb et al. [23]	2022 Australia	Elective surgery, mixed (excluding cardiac and neurosurgery)	748	
Intra‐operative only	Myles [49]	1992 Australia	General surgery	52	
	Hughes et al. [42]	1994 UK	Elective surgery, mixed; women only	122	
	Myles et al. [50]	1996 Australia	Elective surgery, mixed	363	
Postoperative only	Rigotti et al. [54]	1994 USA	Cardiac (CABG only)	93	
	Stanislaw et al. [59]	1994 USA	Cancer surgery, mixed	26	
	Wewers et al. [66]	1994 USA	Cancer, cardiac and general surgery	80	
	Allen et al. [39]	1996 USA	Cardiac; first‐time CABG only; women only	138	25
	Simon et al. [57]	1997 USA	Elective surgery, mixed	324	
	Griebel et al. [41]	1998 USA	Cancer surgery, mixed	28	
	Warner et al. [64]	2005 USA	Elective surgery, mixed	121	
	Nåsell et al. [52]	2010 Sweden	Acute trauma surgery	105	
	Kadda et al. [17]	2015 Greece	Cardiac, mixed (CABG and other)	500	126
	Krebs et al. [44]	2019 USA	Cancer surgery, mixed	42	
	Matuszewski et al. [6]	2021 USA	Acute trauma surgery	266	

CABG, coronary artery bypass grafting. Where the study population comprises smokers and non‐smokers [17, 22, 34, 43], the number of smokers has been reported ‘number of smokers (n)’. In all other studies, smokers comprised the entire study population.

Most of the outcomes included in the analysis were assessed as being at high risk of bias or noted as raising ‘some concerns’ for bias (online Supporting Information Figure S2). High ratings were mostly due to bias emerging in domain 3 (missing outcome data) or domain 4 (inadequate assessment of outcome). Domain 1 was most likely to be rated at low risk of bias. Funnel plots suggest elements of reporting bias (online Supporting Information Figure S3).

### Smoking cessation at the time of surgery

**Figure 2:**
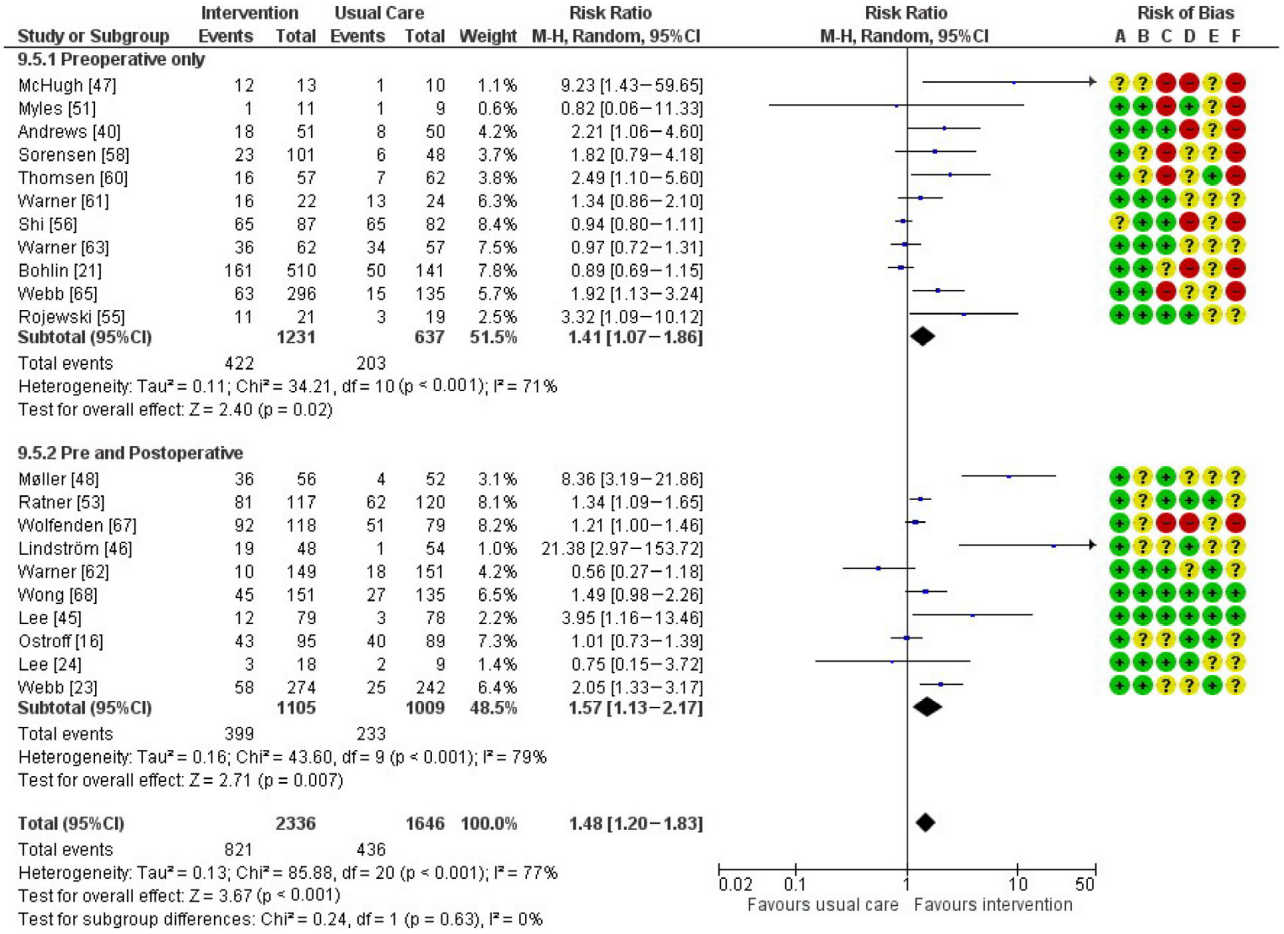
Forest plots for abstinence at the time of surgery. MH, Mantel–Haenszel random effects model. Risk of bias domains: A, randomisation process; B, deviations from intended interventions; C, missing outcomes; D, measurement of the outcome; E, selection of the reported result; F, overall bias. Green circle, low risk of bias; yellow circle, some concerns about bias; red circle, high risk of bias.

Twenty‐one studies reporting smoking cessation at the time of surgery were included in the meta‐analysis (Fig. 2). Overall, peri‐operative smoking cessation interventions were effective in reducing smoking on the day of surgery, RR (95%CI) 1.48 (1.20–1.83), number needed to treat 7.

There was substantial heterogeneity likely contributing to the overall effect estimate when all the studies were combined for this outcome (chi^2^ 85.88, degree of freedom (df) = 20, p < 0.001). A sensitivity analysis excluding the pilot studies had a higher RR (with narrower CIs), 1.52 (1.19–1.96), and with slightly less heterogeneity, although it is still likely to account for the difference in effect estimate (chi^2^ = 75.98, df = 15, p < 0.001). Further sensitivity analysis excluding the pilot studies and the study which examined only a prespecified smoking subgroup yielded similar results, RR (95%CI) 1.47 (1.15–1.87) but, again, with less heterogeneity (chi^2^ = 69.63, df = 14, p < 0.001).

Five studies reporting the day of surgery abstinence outcome had > 20% of their recruited participants drop out: three studies from the pre‐operative only subgroup, and two from the combined pre‐ and postoperative subgroup (online Supporting Information Table S3). In a sensitivity analysis excluding these studies, the overall effect estimate was similar, RR (95%CI) 1.54 (1.20–1.96), chi^2^ = 67.45, df = 15, p < 0.001.

### Smoking cessation at 12 months postoperatively

**Figure 3:**
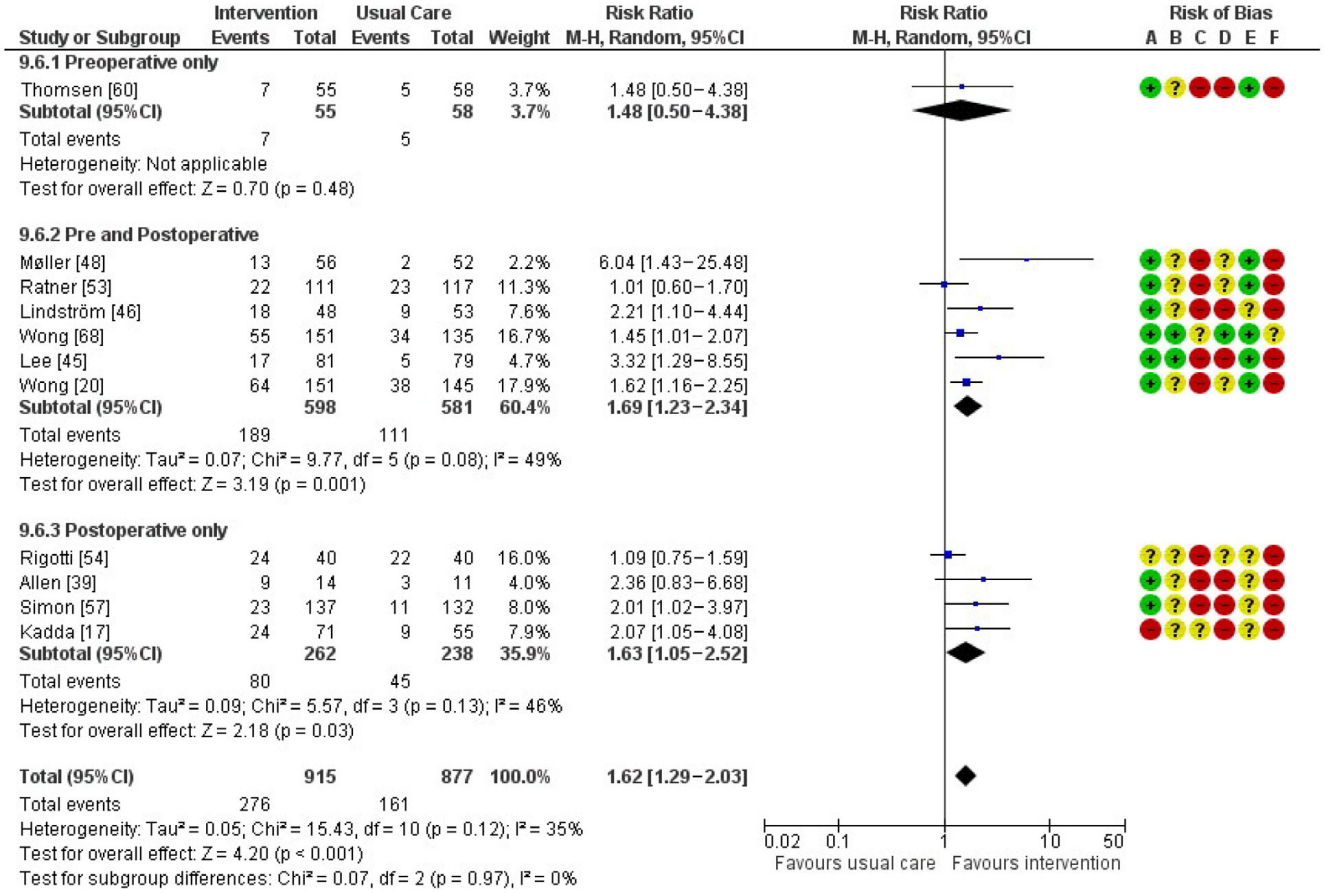
Forest plots for abstinence 12 months postoperatively. MH, Mantel–Haenszel random effects model. Risk of bias domains: A, randomisation process; B, deviations from intended interventions; C, missing outcomes; D, measurement of the outcome; E, selection of the reported result; F, overall bias. Green circle, low risk of bias; yellow circle, some concerns about bias; red circle, high risk of bias.

Eleven studies reporting smoking cessation 12 months after surgery were included in the meta‐analysis (Fig. 3). The overall RR (95%CI) for smoking cessation 12 months postoperatively was 1.62 (1.29–2.03), number needed to treat 9. A sensitivity analysis excluding the studies that focused only on a smoking subgroup of participants yielded a RR (95%CI) of 1.68 (1.30–2.17), with slightly less heterogeneity (chi^2^ = 10.25, df = 7, p = 0.17).

Only one study reporting the 12‐month abstinence outcome was affected by > 20% drop‐out. This study assessed pre‐ and postoperative interventions. In a sensitivity analysis excluding this study, the overall effect estimate was similar, RR (95%CI) 1.70 (1.35–2.14), chi^2^ = 12.88, df = 9, p = 0.17.

### Subgroup analysis by timing of intervention

Of the studies reporting abstinence at the time of surgery, 11 explored the impact of solely pre‐operative interventions, and 10 explored the impact of interventions that combined pre‐ and postoperative measures. The RR (95%CI) for cessation was slightly higher for the combined pre‐ and postoperative subgroup, RR 1.57 (1.13–2.17) than for the solely pre‐operative interventions, RR 1.41 (1.07–1.86), although there was no statistically significant subgroup effect (chi^2^ = 0.24, df = 1, p = 0.63).

Of the studies that reported smoking cessation at 12 months postoperatively, one examined pre‐operative interventions only, six examined interventions that combined pre‐ and postoperative measures, and four examined postoperative interventions only. Three of the four postoperative studies looked only at a prespecified subgroup of smoking participants [17, 39, 54]. Only one pre‐operative study reported this outcome, RR (95%CI) 1.48 (0.5–4.38), the CI including the possibility that there is no significant difference [60]. The RRs (95%CI) for the other two temporal subgroups (postoperative only, and both pre‐ and postoperative interventions) were similar at 1.63 (1.05–2.52) and 1.69 (1.23–2.34), respectively, as was the overall RR for this outcome. There was no statistically significant subgroup effect (chi^2^ = 0.07, df = 2, p = 0.97).

### Subgroup analysis by intensity of intervention

Intensive smoking cessation interventions appeared to increase the likelihood of smoking cessation at 12 months, RR (95%CI) 2.35 (1.48–3.72), and at the time of surgery, the corresponding value being 4.35 (0.90–21.14), although at this time‐point the CI included the possibility of no difference due to the influence of one outlying study [16] (online Supporting Information Figure S4). Short interventions increased the likelihood of smoking cessation at the time of surgery and at 12 months, RRs (95 CI) 1.33 (1.09–1.62) and 1.45 (1.10–1.91), respectively. There were statistically significant subgroup differences at the time of surgery (chi^2^ = 2.14, df = 2, p = 0.34) and at 12 months (chi^2^ = 3.12, df = 2, p = 0.21). Across all time‐points, the likelihood of successfully stopping smoking was highest for intensive smoking cessation interventions.

## Discussion

Our meta‐analysis found that more people who were allocated randomly to peri‐operative tobacco cessation interventions successfully quit smoking at the time of surgery when compared with people allocated to usual care. They were most likely to quit with intervention programmes that extended across the pre‐operative and the postoperative time periods, and for intensive smoking cessation interventions. This implies that more intensive programmes, encompassing all stages of the peri‐operative pathway, may be the most effective strategy to help our patients quit tobacco smoking in the short and long term.

The 2014 Cochrane review of pre‐operative smoking cessation interventions classified two trials as “intensive behaviour support programmes”; both showed evidence of large effects on smoking cessation [15]. The intensity classification used by Thomsen et al. [15] was not directly applicable to our review as it was focused on pre‐operative interventions only. We adopted the more recent intensity classification from Rasmussen et al. [37] and divided our studies into three categories: intensive smoking cessation interventions (meeting set criteria including four or more counselling sessions, medication and provision of self‐help material); short interventions (other behavioural interventions not meeting the intensive criteria); and other interventions (including studies of non‐nicotine replacement pharmacotherapy alone). While most of the studies included in our review are classified as short interventions, our meta‐analysis also suggests that intensive interventions could be the most effective measures across the entire duration of the peri‐operative period (online Supporting Information Figure S4).

Our review included trials of the most up‐to‐date tobacco cessation interventions such as e‐cigarettes assessed in patients awaiting surgery. It also contained interventions that were undertaken at any time‐point throughout the peri‐operative period, and more trials examining long‐term smoking cessation outcomes than earlier reviews. The definition of the peri‐operative period we applied is from the Royal College of Anaesthetists' Centre for Peri‐operative Care, where the peri‐operative period encompasses the care of patients from the moment of contemplation of surgery until full recovery [28]. An increasing proportion of surgery is urgent or time‐critical, and assessed against quality standards which specify short times between referral and surgery. Patients being treated under these circumstances are often those same surgical populations with a high prevalence of tobacco smoking, such as patients referred for acute vascular surgery [6]. Limited time between pre‐operative assessment and surgery can also be a problem in routinely scheduled elective care. Studies by Shi et al. [56] and Warner et al. [62, 64] were undertaken in centres where pre‐operative assessment clinic occurs only on the day before surgery. In these circumstances an intensive, but solely pre‐operative, intervention may not be practical, but the pre‐operative period represents only a fraction of the patient's overall peri‐operative care. Previous reviews on this topic did not include studies with intervention protocols that start during or after surgery [15]. We consider this to be a missed opportunity to analyse, and potentially harness, the impact of a group of interventions with great practical relevance. Postoperative interventions are also important when considering the role of surgery as a “teachable moment” [9], aiming to improve longer‐term abstinence and morbidity related to tobacco smoking and not necessarily to the surgery itself. By including interventions taking place at all stages of the peri‐operative pathway, our review identified numerous effective tobacco cessation interventions (across a wide variety of surgical populations) which included only a short pre‐operative component, or which were primarily focused on the postoperative period. The results from this review support the delivery of tobacco cessation interventions at the time of surgery from the perspective of broader public health, as well as from that of a peri‐operative physician.

The outcomes assessed in most of our included studies had a high risk of bias overall. This was predominantly due to judgements in domain 3 (bias due to missing data) and domain 4 (bias due to inadequate outcome assessment). Missing data due to participants dropping out, or being otherwise lost to follow‐up, were a particular problem for studies which relied on patient‐reported outcomes assessed at longer time‐points. It is convention that, in clinical trials of smoking cessation, any participants lost to follow‐up are assumed to still be smoking [36]. High risk of bias assessments in domain 4 were predominantly due to trials relying on patient self‐reporting of smoking abstinence, rather than a biochemically verified outcome using expiratory carbon monoxide monitoring, or cotinine or anabasine measurements from urine or saliva. A judgement of ‘some concerns for bias’ was made where trials undertook biochemical validation on only a proportion of participants, or where the authors assessed expiratory carbon monoxide but did not use these measurements to categorise abstinence at the individual participant level and reported only mean values. Outcomes were also judged at ‘some risk of bias’ if urine or salivary tests were sent to participants by post; the trials were not blinded, and hence this method of remote biochemical validation was open to manipulation.

A further limitation and important descriptive finding from our review is the heterogeneity of the included trials at all stages, but particularly with respect to outcomes. The Core Outcome Measures in Effectiveness Trials (COMET) initiative seeks to tackle heterogenous outcome reporting by promoting standardised collections of outcomes, known as ‘core outcome sets’ [69]. A core outcome set was put forward for trials of smoking cessation in 2005 [36]. The variety of outcomes assessed by even the most recent studies identified in this review show that such an outcome set is not yet in common use practically. We acknowledge the increased resource demand required to biochemically verify smoking cessation outcomes. While this is not always possible in smaller studies, it does reduce the risk of bias. The selection of participants is another noteworthy source of heterogeneity in this review. Most of the studies examined patients undergoing mixed elective surgery, but some trials looked only at particular surgical groups (Tables 1 and 2, online Supporting Information Table S5) with their own characteristics. The definition of a ‘smoker’ varied: for example, Rigotti et al. included “recent quitters” in their eligibility criteria due to “high rates of relapse” [54]. More concerningly, some studies included only patients that they assessed as ‘ready to quit smoking’, which greatly limits the generalisability of their findings [50, 57]. This is a potential problem underlying the low rates of recruitment to smoking cessation trials: the patients willing to participate may be more likely to want to change. The patients not recruited to smoking cessation trials may be in most need of support, but the conclusions drawn from the evidence may not apply to them. The variety of participants assessed increases the external validity of the review, but due to the resulting heterogeneity, it reduces the overall quality of the evidence.

Enrolment of eligible patients was a problem for the trials included in this review. The number of eligible patients was not reported in five of the included studies, and eight studies explicitly stated that they suffered difficulties in enrolling patients [22, 23, 43, 46, 49, 50, 51, 54]. Two trials were forced to change their protocol: Myles et al. changed their primary outcome, while Kehlet et al. expanded the trial inclusion criteria [43, 51]. Despite these changes, the Kehlet et al. trial closed without reporting secondary outcome data. Loss to follow‐up is also a common issue in smoking cessation trials: seven studies included in this review had > 20% loss to follow‐up at the conclusion of the study, with a range of 0% to 79%. Difficulties with enrolment and adherence are commonly reported in clinical trials investigating tobacco cessation, while intervention adherence positively correlates with abstinence [70]. The frequency of such problems emphasises the importance of embedding qualitative work within clinical trials of behaviour change interventions, to ensure acceptability of the intervention to participants. It also underscores the need to contemporaneously explore the perspectives of tobacco smokers who decline to participate or withdraw from studies. This will allow trials to promptly adapt, minimise dropout and missing outcomes, and improve future research design.

Twenty‐three of the 38 studies included in our review are more than 10 years old, and 11 are more than 20 years old. The older studies included in our meta‐analysis tend to look at postoperative interventions only. This may reflect changing attitudes towards pre‐emptive risk factor modification before surgery and the emergence of peri‐operative medicine. It is likely that many of the surgical, anaesthetic and smoking cessation practices experienced by earlier study populations are different to those of the present day. Were it not for similar results from contemporaneous studies included in our review, it would be reasonable to question the applicability of any conclusions drawn from these older studies to the modern‐day peri‐operative population. In addition, the inclusion of older studies in the meta‐analysis increased heterogeneity: a sensitivity analysis excluding studies more than 20 years old still provides evidence of the effectiveness of peri‐operative smoking cessation interventions at both time‐points, although the overall effect estimates are smaller, the CIs are narrower, and there is less heterogeneity than when all the studies are included (online Supporting Information Table S6). The accepted standards for reporting trials have also become more rigorous with time. Using the modern Risk of Bias‐2 tool in our analysis, many of the older trials have been judged to be at some risk, or at high risk, of bias in one or more domains. This does not mean that they were undertaken with any less scientific diligence, only that there is insufficient information available to retrospectively assess these earlier studies to the reporting standards of the present day. For example, in the past it was less common to publish a protocol or statistical analysis plan, leading to greater risk of bias in domain 5 of the Risk of Bias tool. Published protocols for future randomised controlled trials in peri‐operative smoking cessation are available in online Supporting Information Table S7.

In this systematic review and meta‐analysis, we have summarised the evidence to show that peri‐operative tobacco cessation interventions lead to increased abstinence, both at the time of surgery and 12 months postoperatively. The overall quality of evidence is moderate due to substantial heterogeneity permeating all aspects of trial design. More intensive peri‐operative smoking cessation interventions that continue into the postoperative period are likely to be more effective. Further studies are needed to incorporate the latest evidence‐based tobacco cessation interventions into existing surgical pathways, and to properly account for the particular demands of the acute surgical setting. They should use standardised outcome measures to reduce heterogeneity. More research into patient perspectives is needed to inform the delivery of peri‐operative tobacco cessation interventions that are acceptable to patients and that can be incorporated to improve outcomes in contemporary surgical care.

## Supporting information


**Figure S1.** Literature search strategy.
**Figure S2.** ROB2 judgements for the reported abstinence outcome of all included studies.
**Figure S3.** Funnel plots for each meta‐analysis outcome: (a) abstinence at the time of surgery, and (b) abstinence at 12 months postoperatively.
**Figure S4.** Forest plots of (a) time of surgery abstinence (b) sensitivity analysis for time of surgery abstinence excluding the outlier, Ostroff et al. and (c) 12‐month abstinence.

**Appendix S1.** Data extraction form.

**Table S1.** Excluded studies from full‐text review
**Table S2.** Pilot and subgroup studies.
**Table S3.** Detailed description of interventions and outcomes for each included study.
**Table S4.** Studies with >20% loss to follow up and < 80% eligible recruitment.
**Table S5.** Characteristics of the randomised controlled trials included in the systematic review.
**Table S6.** Results of a sensitivity analysis excluding studies more than 20 years old.
**Table S7.** Published protocols for future trials.
