# Supplementary material for: Peri‐operative tobacco cessation interventions: a systematic review and meta‐analysis
Source: Anaesthesia. 2023 Sep 1;78(11):1393–408. doi: 10.1111/anae.16120 (PMC10952322; doi:10.1111/anae.16120)

Figure S1 Literature Search Strategy

This search has been developed with the help of an information specialist [SR]

Ovid MEDLINE(R) ALL <1946 to July 07, 2022>

1 smoke*.ti,ab. 147122

2 "Tobacco Use Disorder"/ 12064

3 exp Smoking/ 158059

4 Tobacco/ or exp Tobacco Products/ 42941

5 tobacco.ti,ab. 110972

6 exp Smoking Devices/ 15555

7 (cigarette* or "e-cigarette*" or "electronic cigarette*" or vape* or vap?ing or cigar?).ti,ab. 80944

8 exp pipe smoking/ or exp tobacco smoking/ or vaping/ 153721

9 1 or 2 or 3 or 4 or 5 or 6 or 7 or 8 319299

10 (reduc* or quit* or stop* or abstin* or abstain* or cessat* or prevent* or consum* or program or intervention or "cut* down" or "cut-down" or "cut back" or "cut-back" or "cold turkey" or control* or schedul*).ti,ab. 9267831

11 9 and 10 167244

12 Smoking Cessation/ 31670

13 "Tobacco Use Cessation"/ 1386

14 exp "Tobacco Use Cessation Devices"/ 2355

15 ((Nicotine or tobacco) adj5 (replacement or cessation or patch* or "chewing gum" or inhalator* or tablet* or "oral strip*" or lozenge* or spray*)).ti,ab. 10237

16 Varenicline/ 1478

17 (Varenicline or champix).ti,ab. 1939

18 Bupropion/ 3235

19 (Bupropion or Zyban OR Amfebutamone).ti,ab. 4674

20 12 or 13 or 14 or 15 or 16 or 17 or 18 or 19 40785

21 11 or 20 180369

22 (surger* or surgical or operation or operativ* or an?esthesia or "post-operat*" or postoperat* or "pre-operat*" or preoperat* or "peri-operat*" or perioperat* or "post-surg*" or postsurg* or "pre-surg*" or presurg* or "peri-surg*" or perisurg*).ti,ab. 2730098

23 exp Postoperative complication/ 595918

24 exp Preoperative care/ 72452

25 22 or 23 or 24 2967693

26 21 and 25 8458

27 exp animals/ not exp humans/ 5024857

28 26 not 27 8213

Figure S2 ROB2 judgements for the reported abstinence outcome of all included studies

Study ID	Outcome	D1	D2	D3	D4	D5	Overall
Allen 1996	Smoking prevalence at 12 months postoperatively	+	!	-	-	!	-
Andrews 2006	Abstinence at time of surgery	+	+	+	-	!	-
Bohlin 2020	Abstinence at time of surgery	+	+	!	-	!	+
Griebel 1998	Abstinence 6 weeks post discharge	!	-	-	+	!	-
Hughes 1994	Abstinence at 4 weeks postoperatively	!	+	-	-	!	-
Kadda 2015	Abstinence at 12 months postoperatively	-	!	!	-	!	-
Lauridesen 2022	Abstinence at 30 days (alcohol and smoking)	+	!	+	+	+	!
Lauridesen 2022	Abstinence at 12 months (alcohol and smoking)	+	-	-	+	+	-
Lee 2013	Abstinence at time of surgery	+	+	+	+	+	+
Lee 2013	Abstinence at 30 days postoperatively	+	+	-	-	+	-
Lee 2013	Abstinence at 12 months postoperatively	+	+	-	-	+	-
Lee 2018	Abstinence at time of surgery	+	+	+	+	!	!
Lindstrom 2008	Abstinence 3 weeks postoperatively	+	!	!	+	!	!
Lindstrom 2008	Abstinence at 12 months postoperatively	+	!	-	-	!	-
Matuszewski 2021	Abstinence 6 weeks post injury	+	!	-	+	!	-
Matuszewski 2021	Abstinence 3 months post injury	+	!	-	+	!	-
Matuszewski 2021	Abstinence 6 months post injury	+	!	-	+	!	-
McHugh 2001	Abstinence at time of surgery	!	!	-	-	!	-
Moller 2002	Abstinence at time of surgery	+	!	+	!	!	-
Moller 2002	Abstinence at 12 months postoperatively	+	!	-	!	+	-
Myles 1992	Abstinence at 1 month postoperatively	+	+	+	-	!	-
Myles 1992	Abstinence at 6 months postoperatively	+	+	+	-	!	-
Myles 1996	Abstinence at 6 months postoperatively	+	+	-	+	!	-
Myles 1996	Abstinence at 2 months postoperatively	+	+	-	-	!	-
Myles 2004	Abstinence at time of surgery	+	+	-	+	!	-
Myles 2004	Abstinence at 6 months postoperatively	+	+	-	+	!	-
Myles 2004	Abstinence at 6 weeks postoperatively	+	+	-	+	!	-
Myles 2004	Abstinence at 3 weeks postoperatively	+	+	-	+	!	-
Nasell 2010	Abstinence at 2-3 weeks postoperatively	+	+	-	-	!	-
Nasell 2010	Abstinence at 6-12 weeks postoperatively	+	!	-	-	!	-
Ostroff 2014	Abstinence at time of surgery	+	!	!	+	+	!
Ostroff 2014	Abstinence at 3 months postoperatively	+	!	!	+	+	!
Ostroff 2014	Abstinence at 6 months postoperatively	+	!	!	+	+	!
Ratner 2004	Abstinence at time of surgery	+	!	+	+	+	+
Ratner 2004	Abstinence at 6 months postoperatively	+	!	-	!	+	-
Ratner 2004	Abstinence at 12 months postoperatively	+	!	-	!	+	-
Rigotti 1994	Abstinence at 12 months postoperatively	!	!	-	!	!	-
Rigotti 1994	Abstinence at 5.5 year postoperatively	+	!	-	!	!	-
Rigotti 1994	Continuous abstinence at 12 months postoperatively	+	!	-	-	!	-
Rigotti 1994	Continuous abstinence at 5.5 years postoperatively	+	!	-	-	!	-
Rojewski 2021	Abstinence at time of surgery	+	+	+	+	!	!
Rojewski 2021	Abstinence at 30 days postoperatively	+	+	-	-	!	-
Simon 1997	Abstinence at 12 months postoperatively	+	!	-	-	!	-
Simon 1997	Abstinence at 6 months postoperatively	+	!	-	-	!	-
Simon 1997	Abstinence at 12 months postoperatively	+	!	-	-	!	-
Sorensen 2007	Abstinence at time of surgery	+	!	-	!	!	-
Sorensen 2007	Abstinence at time of suture removal	+	!	-	!	!	-
Sorensen 2007	Abstinence at 3 months postoperatively	+	!	-	!	!	-
Shi 2013	Abstinence at time of surgery	!	+	+	-	!	-

+ Low risk
! Some concerns
- High risk

D1 Randomisation process
D3 Missing outcome data
D4 Measurement of the outcome
D5 Selection of the reported result

Thomsen 2010	Abstinence at time of surgery	+	!	-	!	+	-
Thomsen 2010	Abstinence at 12 months postoperatively	+	!	-	-	+	-
Warner 2005	Abstinence at 30 days postoperatively	+	+	-	!	!	-
Warner 2005	Continuous abstinence at 30 days postoperatively	+	+	-	!	!	-
Warner 2005	Abstinence at 6 months postoperatively	+	+	-	!	!	-
Warner 2005	Continuous abstinence at 6 months postoperatively	+	+	-	!	!	-
Warner 2011	Abstinence at time of surgery	+	+	+	!	+	-
Warner 2011	Abstinence at 30 days postoperatively	+	+	-	-	+	-
Warner 2011	Continuous abstinence at 30 days postoperatively	+	+	-	-	+	-
Warner 2011	Abstinence at 90 days postoperatively	+	+	-	-	+	-
Warner 2011	Continuous abstinence at 90 days postoperatively	+	+	-	-	+	-
Warner 2012	Abstinence at time of surgery	+	+	+	!	!	!
Warner 2015	Abstinence at time of surgery	+	+	+	!	!	!
Warner 2015	Abstinence at 30 days postoperatively	+	+	-	-	!	-
Webb 2020	Abstinence at time of surgery	+	+	-	!	!	-
Webb 2022	Abstinence at time of surgery	+	+	!	!	+	!
Webb 2022	4 week continuous abstinence before surgery	+	+	!	-	+	-
Wolfenden 2005	Abstinence at time of surgery	+	!	-	-	!	-
Wolfenden 2005	Abstinence at 3 months postoperatively	+	!	-	-	!	-
Wong 2012	Abstinence at time of surgery	+	+	+	+	+	+
Wong 2012	Abstinence at 12 months postoperatievly	+	+	!	+	+	!
Wong 2012	Abstinence at 3 months postoperatively	+	+	!	+	+	!
Wong 2012	Abstinence at 6 months postoperatively	+	+	!	+	+	!
Wong 2017	Abstinence at time of surgery	+	!	+	+	+	!
Wong 2017	Abstinence at 12 months postoperatively	+	!	-	!	+	-
Wong 2017	Abstinence at 1 month postoperatively	+	!	-	!	+	-
Wong 2017	Abstinence at 3 months postoperatively	+	!	-	!	+	-
Wong 2017	Abstinence at 6 months postoperatively	+	!	-	!	+	-

Figure S3 Funnel plots for each meta-analysis outcome: (a) abstinence at the time of surgery, and (b) abstinence at 12 months postoperatively. The standard error of the logarithm of the risk ratio, SE(log[RR]), is plotted against the effect size for all studies included in the meta-analysis. Studies are shown within their temporal subgroups (preoperative only, pre and postoperative, postoperative only).

a. Abstinence at the time of surgery

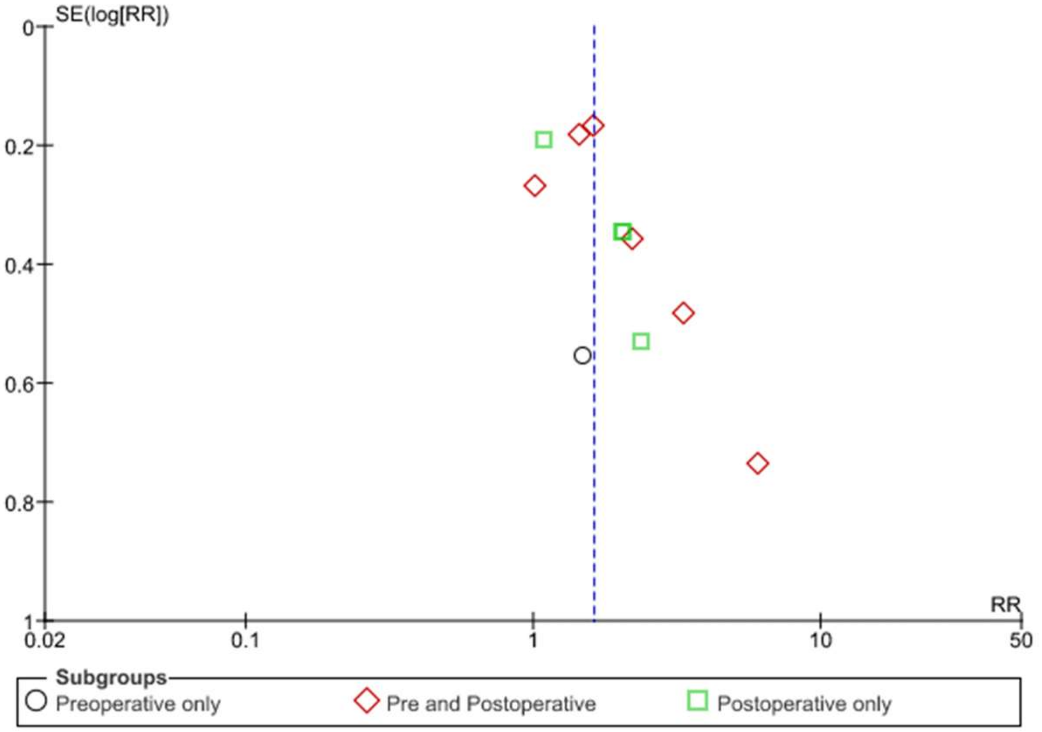

b. Abstinence at 12 months postoperatively

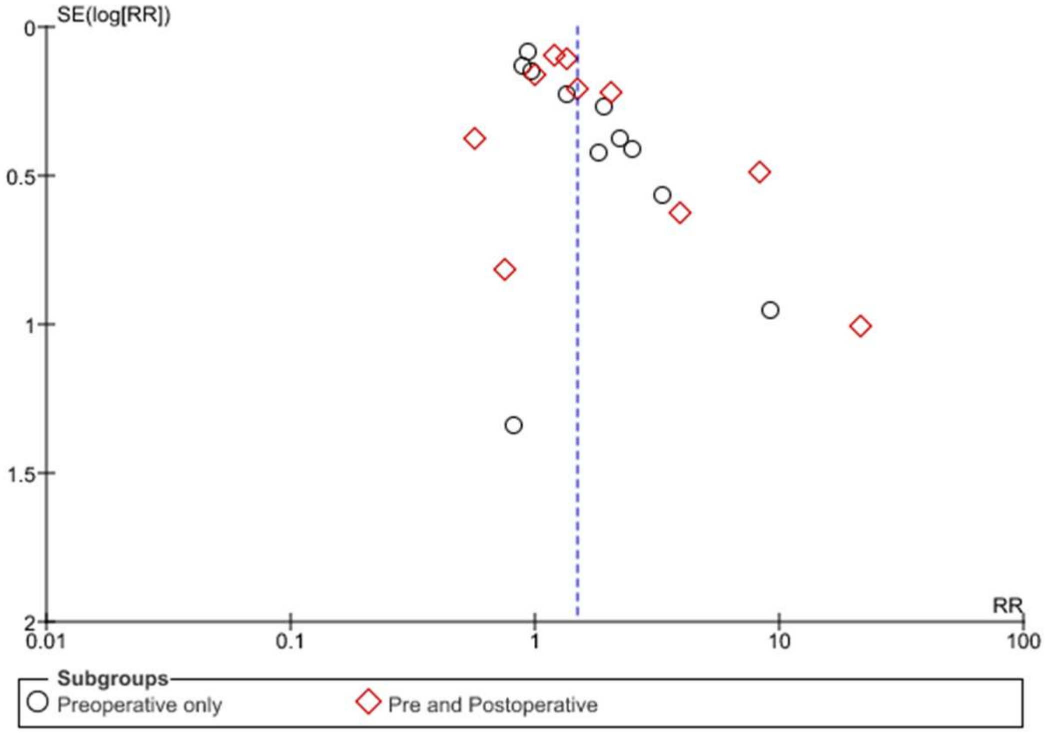

Figure S4 Forest plots of (a) time of surgery abstinence, (b) sensitivity analysis for time of surgery abstinence excluding the outlier, Ostroff et al., and (c) 12-month abstinence. For each outcome, studies are pooled overall, and by intervention intensity subgroup. ISCI, intensive smoking cessation intervention; SI, short intervention; other, other intervention, including non-nicotine-replacement pharmacotherapy alone.

Reference

Rasmussen M, Lauridsen SV, Pedersen B, Backer V, Tønnesen H. Intensive versus short face-to-face smoking cessation interventions: a meta-analysis. European Respiratory Review 2022; **31**: 220063.

a

	Intervention		Usual Care			Risk Ratio
**Study or Subgroup**	**Events**	**Total**	**Events**	**Total**	**Weight**	**M-H, Random, 95% CI**
**9.9.1 ISCI**						
Moller 2002	36	56	4	52	3.1%	8.36 [3.19, 21.86]
Lindstrom 2008	19	48	1	54	1.0%	21.38 [2.97, 153.72]
Ostroff 2014	43	95	40	89	7.3%	1.01 [0.73, 1.39]
Rojewski 2021	11	21	3	19	2.5%	3.32 [1.09, 10.12]
**Subtotal (95% CI)**		**220**		**214**	**13.9%**	**4.35 [0.90, 21.14]**
Total events	109		48			
Heterogeneity: Tau² = 2.24; Chi² = 35.27, df = 3 (P < 0.00001); I² = 91%						
Test for overall effect: Z = 1.83 (P = 0.07)						
**9.9.2 SI**						
McHugh 2001	12	13	1	10	1.1%	9.23 [1.43, 59.65]
Myles 2004	1	11	1	9	0.6%	0.82 [0.06, 11.33]
Ratner 2004	81	117	62	120	8.1%	1.34 [1.09, 1.65]
Wolfenden 2005	92	118	51	79	8.2%	1.21 [1.00, 1.46]
Andrews 2006	18	51	8	50	4.2%	2.21 [1.06, 4.60]
Sorensen 2007	23	101	6	48	3.7%	1.82 [0.79, 4.18]
Thomsen 2010	16	57	7	62	3.8%	2.49 [1.10, 5.60]
Warner 2011	10	149	18	151	4.2%	0.56 [0.27, 1.18]
Wong 2012	45	151	27	135	6.5%	1.49 [0.98, 2.26]
Lee 2013	12	79	3	78	2.2%	3.95 [1.16, 13.46]
Shi 2013	65	87	65	82	8.4%	0.94 [0.80, 1.11]
Warner 2015	36	62	34	57	7.5%	0.97 [0.72, 1.31]
Lee 2018	3	18	2	9	1.4%	0.75 [0.15, 3.72]
Bohlin 2020	161	510	50	141	7.8%	0.89 [0.69, 1.15]
Webb 2020	63	296	15	135	5.7%	1.92 [1.13, 3.24]
Webb 2022	58	274	25	242	6.4%	2.05 [1.33, 3.17]
**Subtotal (95% CI)**		**2094**		**1408**	**79.8%**	**1.33 [1.09, 1.62]**
Total events	696		375			
Heterogeneity: Tau² = 0.08; Chi² = 49.55, df = 15 (P < 0.0001); I² = 70%						
Test for overall effect: Z = 2.81 (P = 0.005)						
**9.9.3 Other**						
Warner 2012	16	22	13	24	6.3%	1.34 [0.86, 2.10]
**Subtotal (95% CI)**		**22**		**24**	**6.3%**	**1.34 [0.86, 2.10]**
Total events	16		13			
Heterogeneity: Not applicable						
Test for overall effect: Z = 1.29 (P = 0.20)						
**Total (95% CI)**		**2336**		**1646**	**100.0%**	**1.48 [1.20, 1.83]**
Total events	821		436			
Heterogeneity: Tau² = 0.13; Chi² = 85.88, df = 20 (P < 0.00001); I² = 77%						
Test for overall effect: Z = 3.67 (P = 0.0002)						
Test for subgroup differences: Chi² = 2.14, df = 2 (P = 0.34), I² = 6.4%						

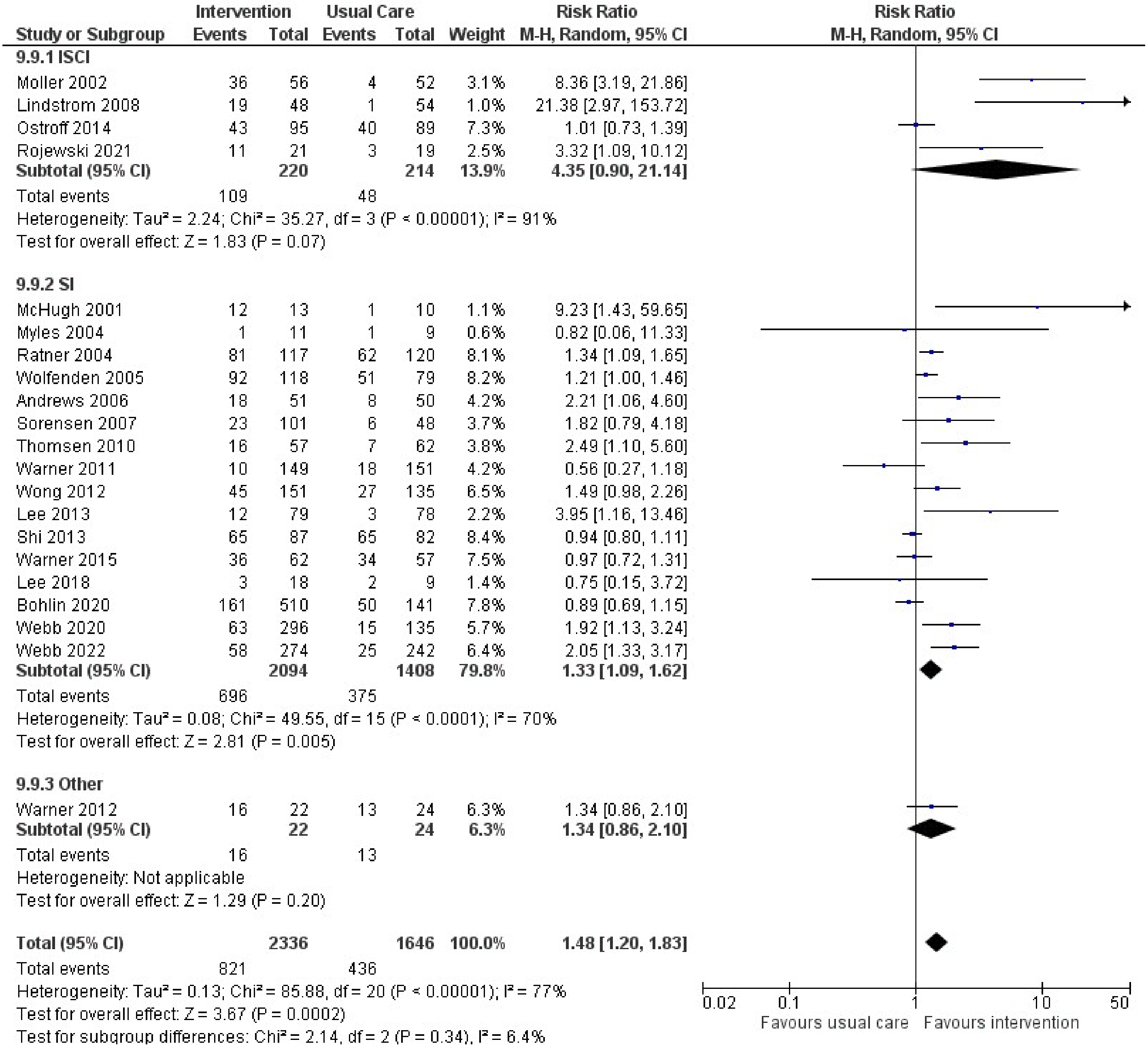

b

Study or Subgroup	Intervention Events	Intervention Total	Usual Care Events	Usual Care Total	Weight	Risk Ratio M-H, Random, 95% CI
**9.9.1 ISCI**						
Moller 2002	36	56	4	52	3.4%	8.36 [3.19, 21.86]
Lindstrom 2008	19	48	1	54	1.1%	21.38 [2.97, 153.72]
Ostroff 2014	43	95	40	89	0.0%	1.01 [0.73, 1.39]
Rojewski 2021	11	21	3	19	2.8%	3.32 [1.09, 10.12]
**Subtotal (95% CI)**		**125**		**125**	**7.4%**	**6.94 [2.73, 17.65]**
Total events	66		8			
Heterogeneity: Tau² = 0.27; Chi² = 3.32, df = 2 (P = 0.19); I² = 40%						
Test for overall effect: Z = 4.07 (P < 0.0001)						
**9.9.2 SI**						
McHugh 2001	12	13	1	10	1.3%	9.23 [1.43, 59.65]
Myles 2004	1	11	1	9	0.7%	0.82 [0.06, 11.33]
Ratner 2004	81	117	62	120	8.5%	1.34 [1.09, 1.65]
Wolfenden 2005	92	118	51	79	8.7%	1.21 [1.00, 1.46]
Andrews 2006	18	51	8	50	4.7%	2.21 [1.06, 4.60]
Sorensen 2007	23	101	6	48	4.1%	1.82 [0.79, 4.18]
Thomsen 2010	16	57	7	62	4.2%	2.49 [1.10, 5.60]
Warner 2011	10	149	18	151	4.6%	0.56 [0.27, 1.18]
Wong 2012	45	151	27	135	7.0%	1.49 [0.98, 2.26]
Lee 2013	12	79	3	78	2.5%	3.95 [1.16, 13.46]
Shi 2013	65	87	65	82	8.8%	0.94 [0.80, 1.11]
Warner 2015	36	62	34	57	7.9%	0.97 [0.72, 1.31]
Lee 2018	3	18	2	9	1.6%	0.75 [0.15, 3.72]
Bohlin 2020	161	510	50	141	8.2%	0.89 [0.69, 1.15]
Webb 2020	63	296	15	135	6.2%	1.92 [1.13, 3.24]
Webb 2022	58	274	25	242	6.9%	2.05 [1.33, 3.17]
**Subtotal (95% CI)**		**2094**		**1408**	**85.8%**	**1.33 [1.09, 1.62]**
Total events	696		375			
Heterogeneity: Tau² = 0.08; Chi² = 49.55, df = 15 (P < 0.0001); I² = 70%						
Test for overall effect: Z = 2.81 (P = 0.005)						
**9.9.3 Other**						
Warner 2012	16	22	13	24	6.8%	1.34 [0.86, 2.10]
**Subtotal (95% CI)**		**22**		**24**	**6.8%**	**1.34 [0.86, 2.10]**
Total events	16		13			
Heterogeneity: Not applicable						
Test for overall effect: Z = 1.29 (P = 0.20)						
**Total (95% CI)**		**2241**		**1557**	**100.0%**	**1.54 [1.23, 1.93]**
Total events	778		396			
Heterogeneity: Tau² = 0.14; Chi² = 86.18, df = 19 (P < 0.00001); I² = 78%						
Test for overall effect: Z = 3.76 (P = 0.0002)						
Test for subgroup differences: Chi² = 11.58, df = 2 (P = 0.003), I² = 82.7%						

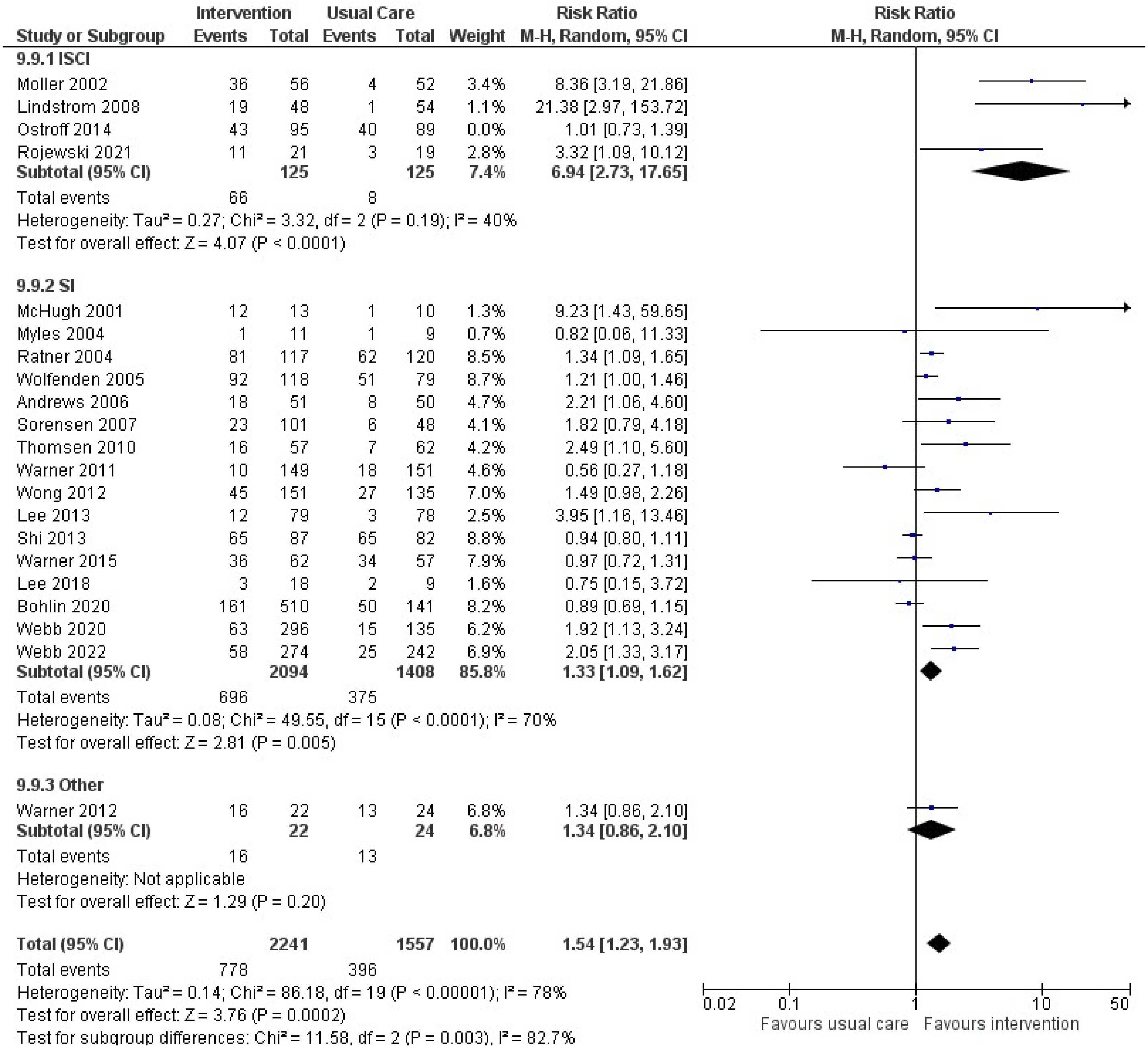

c

Study or Subgroup	Intervention Events	Intervention Total	Usual Care Events	Usual Care Total	Weight	Risk Ratio M-H, Random, 95% CI
**9.10.1 ISCI**						
Simon 1997	23	137	11	132	8.0%	2.01 [1.02, 3.97]
Moller 2002	13	56	2	52	2.2%	6.04 [1.43, 25.48]
Lindstrom 2008	18	48	9	53	7.6%	2.21 [1.10, 4.44]
**Subtotal (95% CI)**		**241**		**237**	**17.8%**	**2.35 [1.48, 3.72]**
Total events	54		22			
Heterogeneity: Tau² = 0.00; Chi² = 1.93, df = 2 (P = 0.38); I² = 0%						
Test for overall effect: Z = 3.63 (P = 0.0003)						
**9.10.2 SI**						
Rigotti 1994	24	40	22	40	16.0%	1.09 [0.75, 1.59]
Allen 1996	9	14	3	11	4.0%	2.36 [0.83, 6.68]
Ratner 2004	22	111	23	117	11.3%	1.01 [0.60, 1.70]
Thomsen 2010	7	55	5	58	3.7%	1.48 [0.50, 4.38]
Wong 2012	55	151	34	135	16.7%	1.45 [1.01, 2.07]
Lee 2013	17	81	5	79	4.7%	3.32 [1.29, 8.55]
Kadda 2015	24	71	9	55	7.9%	2.07 [1.05, 4.08]
**Subtotal (95% CI)**		**523**		**495**	**64.3%**	**1.45 [1.10, 1.91]**
Total events	158		101			
Heterogeneity: Tau² = 0.04; Chi² = 8.90, df = 6 (P = 0.18); I² = 33%						
Test for overall effect: Z = 2.62 (P = 0.009)						
**9.10.3 Other**						
Wong 2017	64	151	38	145	17.9%	1.62 [1.16, 2.25]
**Subtotal (95% CI)**		**151**		**145**	**17.9%**	**1.62 [1.16, 2.25]**
Total events	64		38			
Heterogeneity: Not applicable						
Test for overall effect: Z = 2.85 (P = 0.004)						
**Total (95% CI)**		**915**		**877**	**100.0%**	**1.62 [1.29, 2.03]**
Total events	276		161			
Heterogeneity: Tau² = 0.05; Chi² = 15.43, df = 10 (P = 0.12); I² = 35%						
Test for overall effect: Z = 4.20 (P < 0.0001)						
Test for subgroup differences: Chi² = 3.12, df = 2 (P = 0.21), I² = 35.8%						

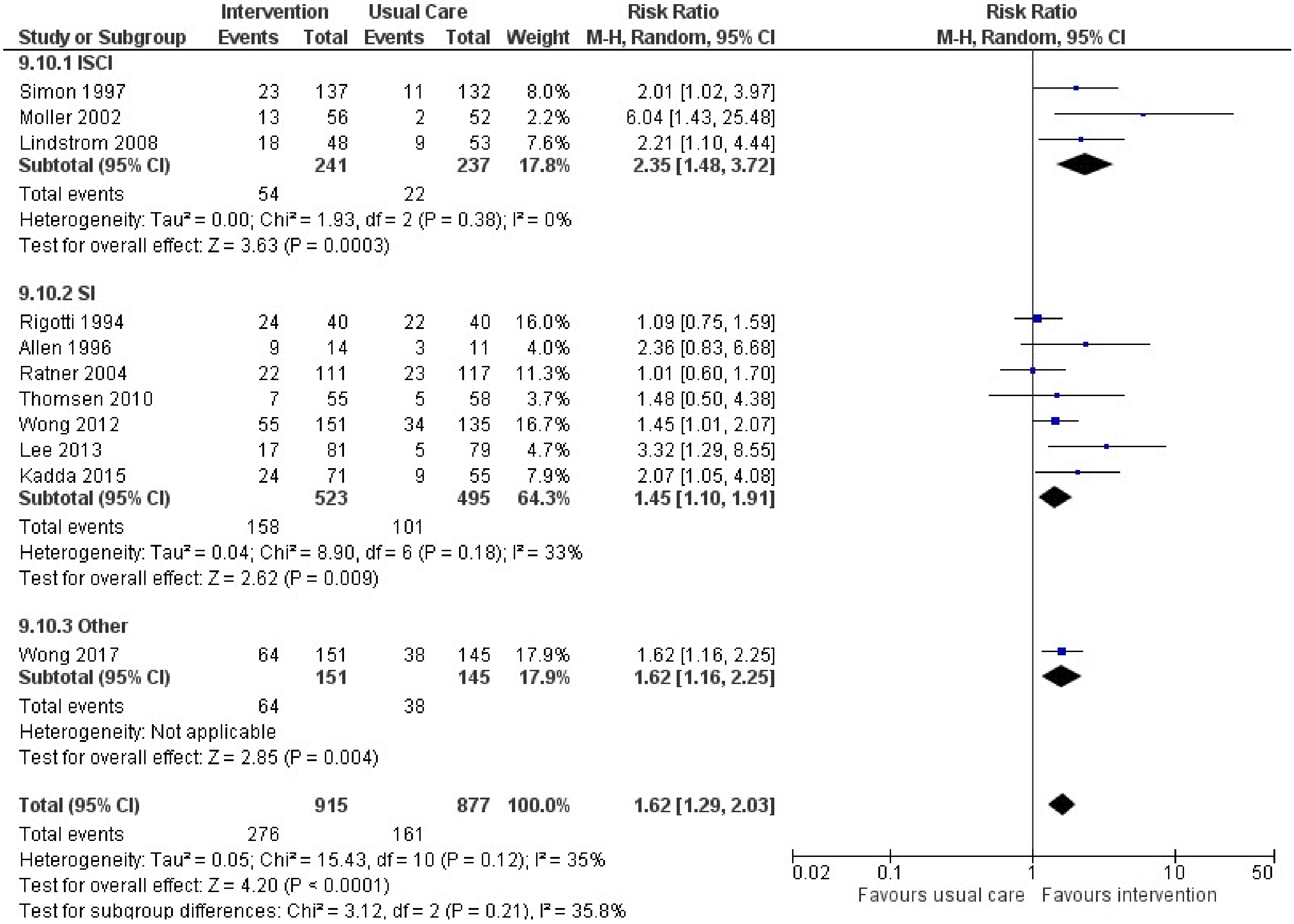

Supplement: Supplementary file 1 — Figure S1. Literature search strategy. Figure S2. ROB2 judgements for the reported abstinence outcome of all included studies. Figure S3. Funnel plots for each meta‐analysis outcome: (a) abstinence at the time of surgery, and (b) abstinence at 12 months postoperatively. Figure S4. Forest plots of (a) time of surgery abstinence (b) sensitivity analysis for time of surgery abstinence excluding the outlier, Ostroff et al. and (c) 12‐month abstinence. [file ANAE-78-1393-s003.pdf]
