# Supplementary material for: Peri‐operative tobacco cessation interventions: a systematic review and meta‐analysis
Source: Anaesthesia. 2023 Sep 1;78(11):1393–408. doi: 10.1111/anae.16120 (PMC10952322; doi:10.1111/anae.16120)
Supplement: Supplementary file 2 — Appendix S1. Data extraction form. [file ANAE-78-1393-s002.pdf]

Data collection form:
Perioperative tobacco-cessation

Information included on this form should be comprehensive, for use in the text of the review, quantitative synthesis, ‘Characteristics of included studies’ table, risk of bias assessment, and statistical analysis.

Notes on using a data extraction form:

- Be consistent in the order and style you use to describe the information.
    - Record any missing information, to make it clear that the information was not found in the study report(s), not that you forgot to extract it e.g.: NOT STATED, NOT APPLICABLE

Contents

- General information
- Methods
- Participants
- Groups (intervention(s) and Control)
- Outcomes (example tables given for dichotomous/continuous/other)
- Other info
- ROB2 assessment

**Numerical Study ID**	
**First Author Surname**	
**Publication Year**	
**Publication Journal**	
**Study author contact details**<br>*(email address, workplace)*	
**Publication type**<br>*(e.g. full report, abstract, letter)*	
Notes	

General Information

**Date form completed**<br>*(dd/mm/yyyy)*	
**Form completed by**	

Notes:

Characteristics of included studies

Methods

	Descriptions as stated in report/paper		Location in text or source *(pg & ¶/fig/table/other)*
**Aim of study** *(e.g. efficacy, equivalence, pragmatic)*			
**Design***(e.g. parallel, crossover, cluster)*			
**Unit of allocation** *(by individuals, cluster/ groups)*			
**Start date**			
**End date**			
**Total study duration** *(from recruitment to last follow-up)*			
**Study funding sources** *(including role of funders)*			
**Possible conflicts of interest** *(for study authors)*			
**Ethical approval needed/ obtained for study**	☐ Yes ☐ No ☐ Unclear		
**Notes:**			

Participants

	Description *(Include comparative information for each intervention or comparison group if available)*		Location in text or source *(pg & ¶/fig/table/other)*
**Population description** *(from which study participants are drawn)*			
**Setting** *(including location [City, Country, Site(s)] and healthcare or social context)*			
**Type of surgery** *(emergent/urgent/elective - specific time targets from referral to surgery if given – specialities and operations if given)*			
**Type of anaesthetic** *(proportion general)*			
**Inclusion criteria**			
**Exclusion criteria**			
**Method of recruitment of participants** *(e.g. phone, mail, clinic patients, voluntary)*			
**Informed consent obtained**	☐ Yes ☐ No ☐ Unclear		
**Total no. randomised**			
**Clusters** *(if applicable, no., type, no. people per cluster)*			
**No. randomised per group** *(specify whether no. people or clusters)*	*Intervention (add column if others)*	*Control*	

**No. missing by group** *(**if by group**, e.g. exclusions & withdrawals, whether or not missing from analysis); if not given by group please state **overall** exclusions & withdrawals, whether or not missing from analysis)*			
**Reasons missing**			
**No. participants moved from one group to another**			
**Reasons moved**			
**Baseline imbalances**			
**Age**			
**Sex** *(proportion male)*			
**Race/Ethnicity**			
**Smoking status** *(how was this categorised [e.g. current, previous, never smokers] and defined)*			
**Method of assessment of smoking status at baseline** *(e.g. patient reported, biochemical validation)*			
**Co-morbidities** *(details of comorbidities given in the demographics, i.e. were the groups similar)*			
**ASA score** *(proportions given)*			

**Any other relevant sociodemographics**		
**Subgroups measured** *(e.g. by age or sex, and was it planned?)*		
**Subgroups reported** *(and was it planned?)*		
**Notes:**		

Intervention/control groups

Copy and paste table for each intervention and control/comparison group, as required

Intervention Group 1

	**Description as stated in report/paper**	**Location in text or source** *(pg & ¶/fig/table/other)*
**Group name** *(from paper e.g. control, intervention)*		
**Theoretical basis for intervention** *(as given by authors, please copy and paste key references)*		
**Description of intervention** *(include sufficient detail for replication e.g. components, here or in specific rows below)*		
**Duration of treatment period**		
**Pre, intra, (and/or) post-operative intervention?**		
**Timing** *(e.g. frequency, duration of each intervention episode, temporal relationship to surgery)*		
**Delivery** *(e.g. mechanism, medium, dose, intensity, fidelity)*		
**Providers** *(no., profession e.g. type of therapist, training)*		

**Co-interventions** *(additional to study intervention e.g. other lifestyle interventions, if given)*		
**Integrity of delivery** *(Was the intervention **delivered** as was originally planned?)*		
**Compliance** *(Did the participants **complete the intervention** in the manner originally planned?)*		
**Notes:**		

Data and analysis

This form includes a blank table for 3 types of outcome:

1. ***Dichotomous*** *(e.g. yes/no, present/absent)*
2. ***Continuous*** *(e.g. height, BP)*
3. ***Other***

Copy and paste the appropriate tables as required to collect data for each outcome in the paper*. Delete those not required.*

If a composite outcome e.g. any complication, is reported, please collect information relating to this

1. Dichotomous outcome

	**Description as stated in report/paper**	**Location in text or source** *(pg & ¶/fig/table/other)*
**Outcome name**		
**Outcome definition and method of assessment** *(with diagnostic criteria if relevant e.g. patient-reported outcome, or biochemical validation of cessation, specifics of test or tool used)*		
**Person measuring/ reporting** *(be specific e.g. patient-reported outcome via survey or described to an investigator)*		
**Time points measured** *(specify whether from start or end of intervention)*		
**Time points reported**		
**Unit of measurement** *(if relevant)*		

**Scales: upper and lower limits** *(indicate whether high or low score is good)*					
**Is outcome/tool validated?**	☐ Yes ☐ No ☐ Unclear		Notes		
**Imputation of missing data** *(e.g. assumptions made for ITT analysis)*					
**Assumed risk estimate** *(e.g. baseline or population risk if noted in Background)*					
**Power** *(e.g. power & sample size calculation, level of power achieved)*					
**Results** *This info is important for the meta-analysis*	Intervention		Comparison		
	No. with event	Total in group	No. with event	Total in group	
**Baseline Data**	Intervention		Comparison		
	No. with event	Total in group	No. with event	Total in group	
**Any other results reported** *(e.g. odds ratio, risk difference, CI or P value)*					
**No. missing participants**					
**Reasons missing**					
**No. participants moved from other group**					
**Reasons moved**					

**Intention to treat, or per-protocol analysis?**			
**Unit of analysis** *(by individuals, cluster/groups or body parts)*			
**Statistical methods used and appropriateness of these** *(e.g. adjustment for correlation)*			
**Reanalysis required?** *(specify, e.g. correlation adjustment)*	☐ Yes ☐ No ☐ Unclear		
**Reanalysis possible?**	☐ Yes ☐ No ☐ Unclear		
**Reanalysed results**			
**Notes:**			

2. Continuous outcome

	**Description as stated in report/paper**	**Location in text or source** *(pg & ¶/fig/table/other)*
**Outcome name**		
**Outcome definition and method of assessment** *(with diagnostic criteria if relevant e.g. patient-reported outcome, or biochemical validation of cessation, specifics of test or tool used)*		

**Person measuring/ reporting** *(be specific e.g. patient-reported outcome via survey or described to an investigator)*			
**Time points measured** *(specify whether from start or end of intervention)*			
**Time points reported**			
**Unit of measurement** *(if relevant)*			
**Scales: upper and lower limits** *(indicate whether high or low score is good)*			
**Is outcome/tool validated?**	☐ Yes ☐ No ☐ Unclear		
**Imputation of missing data** *(e.g. assumptions made for ITT analysis)*			
**Assumed risk estimate** *(e.g. baseline or population risk noted in Background)*			
**Power** *(e.g. power & sample size calculation, level of power achieved)*			
**Post-intervention or change from baseline?**			
**Results**	Intervention	Comparison	

This info is important for the meta-analysis	Mean	SD (or other variance, specify)	No participants	Mean	SD (or other variance, specify)	No participants	
**Any other results reported** *(e.g. mean difference, CI, P value)*							
**No. missing participants**							
**Reasons missing**							
**No. participants moved from other group**							
**Reasons moved**							
**Intention to treat, or per-protocol analysis?**							
**Unit of analysis** *(individuals, cluster/ groups or body parts)*							
**Statistical methods used and appropriateness of these** *(e.g. adjustment for correlation)*							
**Reanalysis required?** *(specify)*	☐ Yes ☐ No ☐ Unclear						
**Reanalysis possible?**	☐ Yes ☐ No ☐ Unclear						
**Reanalysed results**							
**Notes:**							

3. Other Outcome Type

	Description as stated in report/paper		Location in text or source *(pg & ¶/fig/table/other)*
**Outcome name**			
**Outcome definition and method of assessment** *(with diagnostic criteria if relevant e.g. patient-reported outcome, or biochemical validation of cessation, specifics of test or tool used)*			
**Person measuring/ reporting** *(be specific e.g. patient-reported outcome via survey or described to an investigator)*			
**Time points measured** *(specify whether from start or end of intervention)*			
**Time points reported**			
**Unit of measurement** *(if relevant)*			
**Scales: upper and lower limits** *(indicate whether high or low score is good)*			
**Is outcome/tool validated?**	☐ Yes ☐ No ☐ Unclear		
**Imputation of missing data** *(e.g. assumptions made for ITT analysis)*			

**Assumed risk estimate** *(e.g. baseline or population risk noted in Background)*					
**Power** *(e.g. power & sample size calculation, level of power achieved)*					
**Results** *This info is important for the meta-analysis*	Intervention Result	SE or other variance (specify)	Control Result	SE or other variance (specify)	
(timepoint or subgroup, copy rows as needed)					
	Overall Results		SE or other variance		
(timepoint or subgroup, copy rows as needed)					
**Any other results reported** *(e.g. mean difference, CI, P value)*					
**No. missing participants**					
**Reasons missing**					
**No. participants moved from other group**					
**Reasons moved**					
**Intention to treat, or per-protocol analysis?**					
**Unit of analysis** *(individuals, cluster/ groups or body parts)*					
**Statistical methods used and appropriateness of these** *(e.g. adjustment for correlation)*					

**Reanalysis required?** *(specify)*	☐ Yes ☐ No ☐ Unclear		
**Reanalysis possible?**	☐ Yes ☐ No ☐ Unclear		
**Reanalysed results**			
**Notes:**			

Other information

	Description as stated in report/paper	Location in text or source *(pg & ¶/fig/table/other)*
**Key conclusions of study authors**		
**References to other relevant studies**		
**Correspondence required for further study information** *(from whom, what and when)*		
**Notes:**		

Risk of Bias assessment

At this point, please use the excel sheet to carry out the Risk Of Bias 2 assessment on each relevant result in your study.

Use ROB2 Tool https://www.riskofbias.info/welcome/rob-2-0-tool/current-version-of-rob-2 or see [Chapter 8](#) of the Cochrane Handbook.

Crib sheet: https://drive.google.com/file/d/1Q4Fk3HCuBRwIDWTGZa5oH11OdR4Gbhdo/view

	Risk of bias assessment by domain (domains 1-5) (*low/some concerns/high)*					Overall ROB assessment for result	Comments
Outcome/Result	1	2	3	4	5		
