# Supplementary material for: Peri‐operative tobacco cessation interventions: a systematic review and meta‐analysis
Source: Anaesthesia. 2023 Sep 1;78(11):1393–408. doi: 10.1111/anae.16120 (PMC10952322; doi:10.1111/anae.16120)
Supplement: Supplementary file 3 — Table S1. Excluded studies from full‐text review Table S2. Pilot and subgroup studies. Table S3. Detailed description of interventions and outcomes for each included study. Table S4. Studies with >20% loss to follow up and < 80% eligible recruitment. Table S5. Characteristics of the randomised controlled trials included in the systematic review. Table S6. Results of a sensitivity analysis excluding studies more than 20 years old. Table S7. Published protocols for future trials. [file ANAE-78-1393-s001.docx]

**Table S1: Excluded studies from full text review**

|  | **Reason for exclusion** | | | | | |
| --- | --- | --- | --- | --- | --- | --- |
| **Author and title** | **not an RCT** | **wrong PICO** | **mixed surgical cohort** | **mixed smoke cohort** | **protocol** | **Other** |
| Abdelaziz, M.Z., et al., A pre and postoperative rehabilitation programme for lung resection surgery reduces post operative complications and hospital readmission rates. Interactive Cardiovascular and Thoracic Surgery, 2011. 13: p. S40. | 1 |  |  |  |  |  |
| Kerr, A., et al., Rehabilitation for operated lung cancer programme: 18-month outcomes. Interactive Cardiovascular and Thoracic Surgery, 2013. 17: p. S120. | 1 |  |  |  |  |  |
| Abrishami, A., Prevalence of smoking and stage of readiness for smoking cessation in surgical patients. Canadian Journal of Anesthesia, 2010. 57: p. S44-S45. | 1 |  |  |  |  |  |
| Ahc, M., Research: Prehabilitation Can Improve Post-Op Outcomes. Same-Day Surgery, 2020. 44(3): p. N.PAG-N.PAG. | 1 |  |  |  |  |  |
| Akrawi, W. and J.L. Benumof, A pathophysiological basis for informed preoperative smoking cessation counseling. Journal of Cardiothoracic and Vascular Anesthesia, 1997. 11(5): p. 629-640. | 1 |  |  |  |  |  |
| Allen, G., Evidence for practice. Smoking-cessation intervention for surgical patients. AORN Journal, 2005. 81(2): p. 425-426. | 1 |  |  |  |  |  |
| Alvira-Gonzalez, J. and C. Gay-Escoda, Compliance of postoperative instructions following the surgical extraction of impacted lower third molars: A randomized clinical trial. Medicina Oral, Patologia Oral y Cirugia Bucal, 2015. 20(2): p. e224-e234. |  | 1 |  |  |  |  |
| An, et al., Making every contact count: The role of the clinician in smoking cessation during the perioperative period. Clinical Medicine, Journal of the Royal College of Physicians of London, 2020. 20(2): p. E2. | 1 |  |  |  |  |  |
| An, D., et al., Preoperative smoking cessation as part of surgical prehabilitation. Canadian Journal of Anesthesia, 2019. 66(4): p. 476-479. | 1 |  |  |  |  |  |
| Anonymous, The Adjuvant Benefit of Angioplasty in Patients with Mild to Moderate Intermittent Claudication (MIMIC) Managed by Supervised Exercise, Smoking Cessation Advice and Best Medical Therapy: Results from Two Randomised Trials for Stenotic Femoropopliteal and Aortoiliac Arterial Disease. European Journal of Vascular and Endovascular Surgery, 2008. 36(6): p. 680-688. |  | 1 |  |  |  |  |
| Argunova, Y., et al., Adherence to non-pharmacological therapy in patients after coronary artery bypass grafting following different cardiac rehabilitation programs. European Journal of Preventive Cardiology, 2017. 24(1): p. S36. |  | 1 |  |  |  |  |
| Armitage, C.J., Evidence that implementation intentions can overcome the effects of smoking habits. Health Psychology, 2016. 35(9): p. 935-943. |  | 1 |  |  |  |  |
| Ayuso, S.A., et al., Smoking, Obesity, and the Elective Operation. Surgical Clinics of North America, 2021. 101(6): p. 981-993. | 1 |  |  |  |  |  |
| Bahia, S.S., et al., Cardiac rehabilitation versus standard care after aortic aneurysm repair (Aneurysm CaRe): Study protocol for a randomised controlled trial. Trials, 2015. 16(1): p. 162. |  | 1 |  |  |  |  |
| Barassi, G., et al., Preoperative rehabilitation in lung cancer patients: Yoga approach. Advances in Experimental Medicine and Biology, 2018. 1096: p. 19-29. |  | 1 |  |  |  |  |
| Basler HD, Wilcke I. [The changes in smoking habits ofvaso-operated patients.]Medizinische Psychologie1981;7:27 – 43 | 1 |  |  |  |  |  |
| Beaupre, L.A., et al., Impact of a standardized referral to a community pharmacistled smoking cessation program before elective joint replacement surgery. Tobacco Induced Diseases, 2019. 17: p. 1-7. | 1 |  |  |  |  |  |
| Bendtsen, M., C. Linderoth, and P. Bendtsen, Mobile Phone-Based Smoking-Cessation Intervention for Patients Undergoing Elective Surgery: Protocol for a Randomized Controlled Trial. JMIR research protocols, 2019. 8(3): p. e12511. | 1 |  |  |  | 1 |  |
| Berger-Richardson, D., et al., A randomized controlled feasibility study comparing a multimodal prehabilitation protocol to normal care for women undergoing neo-adjuvant chemotherapy for breast cancer. Annals of Surgical Oncology, 2020. 27: p. S162. | 1 | 1 |  |  |  |  |
| Berney, C.R., Enforced smoking cessation programme prior to elective surgery. ANZ journal of surgery, 2020. 90(1): p. 19-20. | 1 |  |  |  |  |  |
| Beyea, S.C., Evidence for practice. Preoperative smoking intervention decreases postoperative complications. AORN Journal, 2002. 76(3): p. 520-520. | 1 |  |  |  |  |  |
| Bjurlin MA, Cohn MR, Kim, DY et al. Brief smoking cessation intervention: a prospective trial in the urology setting. Journal of Urology. 2012. 189: (5) 1843-1849 |  | 1 |  |  |  |  |
| Browning, K.K., et al., Implementing the Agency for Health Care Policy and Research's Smoking Cessation Guideline in a lung cancer surgery clinic. Oncology nursing forum, 2000. 27(8): p. 1248-1254. | 1 |  |  |  |  |  |
| Coca-Martinez, M., et al., Multimodal prehabilitation as strategy for reduction of postoperative complications after cardiac surgery: A randomised controlled trial protocol. BMJ Open, 2020. 10(12): p. e039885. | 1 |  |  |  | 1 |  |
| Coca-Martinez, M., et al., Multimodal Prehabilitation for Peripheral Arterial Disease: Results of an In-Trial Pilot Randomized Controlled Trial. Journal of Vascular Surgery, 2021. 74(5): p. e426-e427. | 1 |  |  | 1 |  |  |
| Coffman, C.R., et al., A short, sustainable intervention to help reduce day of surgery smoking rates among patients undergoing elective surgery. Journal of Clinical Anesthesia, 2019. 58: p. 35-36. | 1 |  |  |  |  |  |
| Conway, T.L., et al., Operation Stay Quit: Evaluation of Two Smoking Relapse Prevention Strategies for Women after Involuntary Cessation during U.S. Navy Recruit Training. Military Medicine, 2004. 169(3): p. 236-242. |  | 1 |  |  |  |  |
| Cummins, S., et al., Nicotine patches and quitline counseling to help hospitalized smokers stay quit: study protocol for a randomized controlled trial. Trials, 2012. 13(1): p. 128-128. |  | 1 |  |  | 1 |  |
| Cummins, S.E., et al., Telephone Intervention for Pregnant Smokers: A Randomized Controlled Trial. American Journal of Preventive Medicine, 2016. 51(3): p. 318-326. |  | 1 | 1 |  |  |  |
| Davis, C., Smoking cessation interventions improved the 12-month smoking cessation rate among patients undergoing non-cardiac surgery. Evidence-based cardiovascular medicine, 1998. 2(1): p. 19. | 1 |  |  |  |  |  |
| Davis, E.M., et al., A Telemedicine Pathway to Increase Tobacco Cessation in Patients Undergoing Total Joint Replacement Surgery. NEJM Catalyst Innovations in Care Delivery, 2021. 2(3): p. 1-1. | 1 |  |  |  |  |  |
| de Jong, B., et al., The safety and efficacy of nicotine replacement therapy in the intensive care unit: a randomised controlled pilot study. Annals of Intensive Care, 2018. 8(1): p. 70. |  | 1 | 1 |  |  |  |
| do Amaral, L.M., et al., Text messaging interventions to support smoking cessation among hospitalized patients in Brazil: a randomized comparative effectiveness clinical trial. BMC research notes, 2022. 15(1): p. 119. |  |  | 1 |  |  |  |
| Engblom, E., et al., Coronary heart disease risk factors before and after bypass surgery: Results of a controlled trial on multifactorial rehabilitation. European Heart Journal, 1992. 13(2): p. 232-237. |  |  |  | 1 |  |  |
| Ferrari, F., et al., Validation of an ERAS protocol in gynecological surgery: Interim analysis of an Italian randomized controlled trial. International Journal of Gynecological Cancer, 2019. 29: p. A40. |  |  |  | 1 |  |  |
| Ferrari F, Forte S, Sbalzer N et al. Validation of an enhanced recovery after surgery protocol in gynecologic surgery: an Italian randomized study. Am J Obstet Gynecol. 2020 Oct;223(4):543.e1-543.e14. doi: 10.1016/j.ajog.2020.07.003. Epub 2020 Jul 8. PMID: 32652064. |  |  |  | 1 |  |  |
| Fonteyn, M.E., A nurse led smoking cessation intervention increased cessation rates after hospital admission for coronary heart disease. Evidence Based Nursing, 2004. 7(2): p. 46-46. | 1 |  |  |  |  |  |
| Froelicher, E.S. and D.J. Christopherson, Women's initiative for nonsmoking (WINS) I: Design and methods. Heart and Lung: Journal of Acute and Critical Care, 2000. 29(6): p. 429-437. |  |  | 1 | 1 | 1 |  |
| Garry, L.L. and U. Atabek, Implementing a smoking cessation program for your patients. Physician assistant (American Academy of Physician Assistants), 1995. 19(11): p. 75-82. | 1 |  |  |  |  |  |
| DeVito Dabbs A, Song MK, Myers BA, et al. A randomized controlled trial of a mobile health intervention to promote self-management after lung transplantation. Am J Transplant. 2016;16:2172–2180 |  |  |  | 1 |  |  |
| Rosenberger EM, DeVito Dabbs AJ, DiMartini AF, Landsittel DP, Pilewski JM, Dew MA. Long-Term Follow-up of a Randomized Controlled Trial Evaluating a Mobile Health Intervention for Self-Management in Lung Transplant Recipients. Am J Transplant. 2017 May;17(5):1286-1293. doi: 10.1111/ajt.14062. Epub 2016 Oct 31. |  |  |  | 1 |  |  |
| Geramita, E.M., et al., Impact of a mobile health intervention on long-term nonadherence after lung transplantation: Follow-up after a randomized controlled trial. Transplantation, 2020: p. 640-651. |  |  |  | 1 |  |  |
| Gibb, C. and R. Fitridge, Smoking and the surgeon: no more excuses. ANZ journal of surgery, 2018. 88(9): p. 815-816. | 1 |  |  |  |  |  |
| Gjeilo, K.H., et al., Smoking cessation after cardiac surgery. A window of opportunity?...7th Annual Cardiovascular Nursing Spring Meeting of the European Society of Cardiology Council on Cardiovascular Nursing and Allied Professions: changing practice to improve care Manchester, UK 23-24 March 2007. European Journal of Cardiovascular Nursing, 2007. 6: p. S8-S8. | 1 |  |  |  |  |  |
| Glasgow, R.E., et al., Evaluating Initial Reach and Robustness of a Practical Randomized Trial of Smoking Reduction. Health Psychology, 2008. 27(6): p. 780-788. |  |  | 1 |  |  |  |
| Glasgow, R.E., et al., Long-term results of a smoking reduction program. Medical Care, 2009. 47(1): p. 115-120. |  |  | 1 |  |  |  |
| Golaghaie, F., et al., Adherence to lifestyle changes after coronary artery bypass graft: Outcome of preoperative peer education. Patient Education and Counseling, 2019. 102(12): p. 2231-2237. |  |  |  | 1 |  |  |
| Goodman, H., et al., A randomised controlled trial to evaluate a nurse-led programme of support and lifestyle management for patients awaiting cardiac surgery: 'Fit for surgery: Fit for life' study. European Journal of Cardiovascular Nursing, 2008. 7(3): p. 189-195. |  |  |  | 1 |  |  |
| Goodney, PP., Spangler, E. L., Newhall, K., et al. Feasibility and pilot efficacy of a brief smoking cessation intervention delivered by vascular surgeons in the Vascular Physician Offer and Report (VAPOR) Trial . Journal of Vascular Surgery. 2017. 65 (4): 1152-1160.e2 |  | 1 | 1 | 1 |  | 1* |
| Granger, C.L., et al., Effect of a postoperative home-based exercise and self-management programme on physical function in people with lung cancer (CAPACITY): Protocol for a randomised controlled trial. BMJ Open Respiratory Research, 2022. 9(1): p. e001189. |  | 1 |  |  |  |  |
| Haddock, J. and C. Burrows, The role of the nurse in health promotion: an evaluation of a smoking cessation programme in surgical pre-admission clinics. Journal of advanced nursing, 1997. 26(6): p. 1098-1110. | 1 |  |  |  |  |  |
| Haile, S., et al., Follow-up after surgical treatment for intermittent claudication (FASTIC): a study protocol for a multicentre randomised controlled clinical trial. BMC nursing, 2020. 19: p. 45. | 1 | 1 |  |  | 1 |  |
| Hajek, P., T.Z. Taylor, and P. Mills, Brief intervention during hospital admission to help patients to give up smoking after myocardial infarction and bypass surgery: Randomised controlled trial. British Medical Journal, 2002. 324(7329): p. 87-89. |  |  | 1 |  |  |  |
| Hartog, J., et al., Heart Rehabilitation in patients awaiting Open heart surgery targeting to prevent Complications and to improve Quality of life (Heart-ROCQ): Study protocol for a prospective, randomised, open, blinded endpoint (PROBE) trial. BMJ Open, 2019. 9(9): p. e031738. | 1 |  |  | 1 | 1 |  |
| Haynen, B.K.M., et al., Impact of coronary revascularization for acute coronary syndrome on smoking cessation. European Heart Journal, 2011. 32: p. 380. |  | 1 |  |  |  |  |
| Hilleman, D.E., S.M. Mohiuddin, and K.A. Packard, Comparison of Conservative and Aggressive Smoking Cessation Treatment Strategies Following Coronary Artery Bypass Graft Surgery. Chest, 2004. 125(2): p. 435-438. | 1 |  |  |  |  |  |
| Hodgson, B., C. Hanrahan, and V. Cuthbertson, Does smoking cessation prior to elective spinal surgery lead to long-term smoking abstinence. The New Zealand medical journal, 2016. 129(1441): p. 101-103. | 1 |  |  |  |  |  |
| Howard, R., et al., Impact of a Regional Smoking Cessation Intervention for Vascular Surgery Patients. Journal of Vascular Surgery, 2021. 74(3): p. e282. | 1 |  |  |  |  |  |
| Iida, H., et al., Preoperative smoking cessation and smoke-free policy in a university hospital in Japan. Canadian Journal of Anesthesia, 2008. 55(5): p. 316-318. | 1 |  |  |  |  |  |
| Julian, D.G., Smoking and coronary artery bypass surgery. British heart journal, 1994. 72(1): p. 9-11. | 1 |  |  |  |  |  |
| Kanova, M., et al., Nicotine replacement therapy in surgical patients. Neuro endocrinology letters, 2021. 42(5): p. 305-311. |  | 1 |  |  |  |  |
| Katz, A., From paper to practice change. AWHONN lifelines / Association of Women's Health, Obstetric and Neonatal Nurses, 2005. 9(3): p. 193-194. | 1 |  |  |  |  |  |
| Kawaguchi, M., M. Ida, and Y. Naito, The role of Perioperative Surgical Home on health and longevity in society: importance of the surgical prehabilitation program. Journal of Anesthesia, 2017. 31(3): p. 319-324. | 1 |  |  |  |  |  |
| Keating, S., Presurgical Tobacco Cessation Counseling. The American journal of nursing, 2016. 116(3): p. 11. | 1 |  |  |  |  |  |
| Kelley, K.E., et al., Impact of a Novel Preoperative Patient-centered Surgical Wellness Program. Annals of surgery, 2018. 268(4): p. 650-656. | 1 |  |  |  |  |  |
| Kerbage, S., et al., Impact of education on secondary prevention in patients undergoing cardiovascular surgery. Circulation, 2012. 125(19): p. e800. | 1 |  |  |  |  |  |
| Kones, R., A. Morales-Salinas, and U. Rumana, Cardiac rehabilitation underutilization: Missed opportunities in comprehensive cardiac care. International Journal of Cardiology, 2019. 292: p. 39-40. | 1 |  |  |  |  |  |
| Lauridsen, S.V., et al., Smoking and alcohol cessation intervention in relation to radical cystectomy: A qualitative study of cancer patients' experiences. BMC Cancer, 2017. 17(1): p. 793. | 1 |  |  |  |  |  |
| Lauridsen, S.V., et al., STOP smoking and alcohol drinking before OPeration for bladder cancer (the STOP-OP study), perioperative smoking and alcohol cessation intervention in relation to radical cystectomy: Study protocol for a randomised controlled trial. Trials, 2017. 18(1): p. 329. | 1 |  |  |  | 1 |  |
| Lin, H.H., et al., Effects of a therapeutic lifestyle-change programme on cardiac risk factors after coronary artery bypass graft. Journal of Clinical Nursing, 2010. 19(1): p. 60-68. | 1 |  |  |  |  |  |
| Moller, A. and H. Tonnesen, Risk reduction: Perioperative smoking intervention. Best Practice and Research: Clinical Anaesthesiology, 2006. 20(2): p. 237-248. | 1 |  |  |  |  |  |
| Moller, A., et al., Pre-operative nicotine replacement and smoking cessation counselling reduce post-operative complications. Evidence-Based Healthcare, 2002. 6(4): p. 190-191. | 1 |  |  |  |  |  |
| Moller, A.M., et al., Preoperative smoking intervention and postoperative complications. Journal fur Anasthesie und Intensivbehandlung, 2002. 9(2): p. 207. | 1 |  |  |  |  |  |
| Molyneux, A., et al., Clinical trial comparing nicotine replacement therapy (NRT) plus brief counselling, brief counselling alone, and minimal intervention on smoking cessation in hospital inpatients. Thorax, 2003. 58(6): p. 484-488. |  |  | 1 |  |  |  |
| Munday, I.T., et al., The effectiveness of pre-operative advice to stop smoking: A prospective controlled trial. Anaesthesia, 1993. 48(9): p. 816-818. | 1 |  |  |  |  |  |
| Murray, E.W., A preoperative smoking intervention decreased postoperative complications in elective knee or hip replacement. Evidence-based nursing, 2002. 5(3): p. 84. | 1 |  |  |  |  |  |
| Newhall, K., et al., Smoking cessation counseling in vascular surgical practice using the results of interviews and focus groups in the Vascular Surgeon offer and report smoking cessation pilot trial. Journal of Vascular Surgery, 2016. 63(4): p. 1011-1017. | 1 |  |  |  |  |  |
| Niu, B., et al., Risk factors for postoperative pulmonary infection in elderly cancer patients and countermeasures. International Journal of Clinical and Experimental Medicine, 2020. 13(6): p. 3883-3891. | 1 |  |  |  |  |  |
| Oldenburg, B., et al., A controlled trial of a behavioral and educational intervention following coronary artery bypass surgery. Journal of Cardiopulmonary Rehabilitation, 1995. 15(1): p. 39-46. |  |  |  | 1 |  |  |
| Patel, Y.S., et al., OA04.01 Move For Surgery - A Novel Preconditioning Program to Optimize Health Before Thoracic Surgery: A Randomized Controlled Trial. Journal of Thoracic Oncology, 2021. 16(10): p. S852-S853. |  | 1 |  |  |  |  |
| Plüss CE, Billing E, Held C, Henriksson P, Kiessling A, Karlsson MR, Wallen HN. Long-term effects of an expanded cardiac rehabilitation programme after myocardial infarction or coronary artery bypass surgery: a five-year follow-up of a randomized controlled study. Clin Rehabil. 2011 Jan;25(1):79-87. doi: 10.1177/0269215510376006. |  | 1 | 1 | 1 |  |  |
| Plüss CE, Karlsson MR, Wallen N, Billing E, Held C. Effects of an expanded cardiac rehabilitation programme in patients treated for an acute myocardial infarction or a coronary artery by-pass graft operation. Clin Rehabil. 2008 Apr;22(4):306-18. |  | 1 | 1 | 1 |  |  |
| Quist-Paulsen, P., P.S. Bakke, and F. Gallefoss, Predictors of smoking cessation in patients admitted for acute coronary heart disease. European journal of cardiovascular prevention and rehabilitation : official journal of the European Society of Cardiology, Working Groups on Epidemiology & Prevention and Cardiac Rehabilitation and Exercise Physiology, 2005. 12(5): p. 472-477. | 1 |  |  |  |  |  |
| Quist-Paulsen, P. and F. Gallefoss, Randomised controlled trial of smoking cessation intervention after admission for coronary heart disease. British Medical Journal, 2003. 327(7426): p. 1254-1257. |  |  | 1 |  |  |  |
| Racelis, M. C. , Lombardo, K. , Verdin, J. Impact of telephone reinforcement of risk reduction education on patient compliance. Journal of vascular nursing. 1998. 16(1):16-20 |  |  | 1 | 1 |  |  |
| Rajaee, S., et al., A Pilot Study of a Standardized Smoking Cessation Intervention for Patients with Vascular Disease. Annals of Vascular Surgery, 2019. 61: p. 91. |  |  | 1 |  |  |  |
| Redfern, J., Expanded cardiac rehabilitation reduces cardiac events over five years. Journal of physiotherapy, 2011. 57(1): p. 57. | 1 |  |  |  |  |  |
| Rigotti, N.A., et al., Efficacy of a smoking cessation program for hospital patients. Archives of Internal Medicine, 1997. 157(22): p. 2653-2660. |  |  | 1 |  |  |  |
| Rissel C, Salmon A, Hughes A. Evaluation of a (pilot)stage-tailored brief smoking cessation intervention amonghospital patients presenting to a hospital pre-admissionclinic.Austr Health Rev2000;23: 83 – 93 | 1 |  |  |  |  |  |
| Rojewski, A.M., et al., Preoperative contingency management intervention for smoking abstinence in cancer patients: trial protocol for a multisite randomised controlled trial. BMJ Open, 2021. 11(6): p. 051226. | 1 |  |  |  | 1 |  |
| Sahin, H., Effects of preoperative smoking cessation on HAM-A sedation scores and intraoperative consumption of anesthetics and fentanyl. Anaesthesia, Pain and Intensive Care, 2017. 21(1): p. 52-58. |  | 1 |  |  |  |  |
| Seijo-Bestilleiro, R., et al., Randomized clinical trial to determine the effectiveness of CO-oximetry and anti-smoking brief advice in a cohort of kidney transplant patients who smoke. International Journal of Medical Sciences, 2020. 17(17): p. 2673-2684. |  | 1 |  |  |  |  |
| Shah, M.V., G. Watkins, and I.P. Latto, The effect of written advice on preoperative cigarette consumption. Annals of the Royal College of Surgeons of England, 1984. 66(6): p. 436-437. | 1 |  |  |  |  |  |
| Sharma, K.K., et al., Non-physician health workers for improving adherence to medications and healthy lifestyle following acute coronary syndrome: 24-month follow-up study. Indian Heart Journal, 2016. 68(6): p. 832-840. |  |  | 1 |  |  |  |
| Siercke, M., et al., Cardiovascular Rehabilitation Increases Walking Distance in Patients With Intermittent Claudication. Results of the CIPIC Rehab Study: A Randomised Controlled Trial. Journal of Vascular Surgery, 2021. 74(6): p. 2118. |  | 1 |  |  |  |  |
| Singh, L.K., et al., Brief Intervention for Tobacco when Diagnosed with Oral Cancer (BITDOC): Study protocol of a randomized clinical trial studying efficacy of brief tobacco cessation intervention, Chhattisgarh, India. Tobacco prevention & cessation, 2020. 6: p. 4. | 1 |  | 1 |  | 1 |  |
| Smaily, H., et al., Smoking cessation intervention for patients with head and neck cancer: A prospective randomized controlled trial. American Journal of Otolaryngology - Head and Neck Medicine and Surgery, 2021. 42(1): p. 102832. |  |  | 1 |  |  |  |
| Sorensen, L.T. and T. Jorgensen, Short-term pre-operative smoking cessation intervention does not affect postoperative complications in colorectal surgery: A randomized clinical trial. Colorectal Disease, 2003. 5(4): p. 347-352. |  | 1 |  |  |  |  |
| Sorensen, L.T., et al., Effect of smoking, smoking cessation, and nicotine patch on wound dimension, vitamin C, and systemic markers of collagen metabolism. Surgery, 2010. 148(5): p. 982-990. |  | 1 |  |  |  |  |
| Smith PM, Reilly KR, Miller NH, DeBusk RF, Taylor CB.Application of a nurse-managed inpatient smoking cessationprogram.Nicotine Tob Res2002;4: 211 – 222 |  |  | 1 |  |  |  |
| Taylor, C.B., et al., A nurse-managed smoking cessation program for hospitalized smokers. American Journal of Public Health, 1996. 86(11): p. 1557-1560. |  |  | 1 |  |  |  |
| Thomsen, T. and M. Finnegan, A preoperative smoking cessation intervention increases postoperative quit rates and may reduce postoperative morbidity. Journal of Bone & Joint Surgery, American Volume, 2011. 93(4): p. 394-394. | 1 |  |  |  |  |  |
| Tobin, M.J., A.F. Suffredini, and A. Grenvik, Short-term effects of smoking cessation. Respiratory Care, 1984. 29(6): p. 641-651. | 1 |  |  |  |  |  |
| Todd, N., A comprehensive smoking cessation programme after surgery increased 1 year quit rates [commentary on Simon JA, Solkowitz SN, Carmody TP, et al. Smoking cessation after surgery. A randomised trial. ARCH INTERN MED 1997 Jun 23;157:1371-6]. Evidence Based Nursing, 1998. 1(2): p. 46-46. | 1 |  |  |  |  |  |
| Tønnesen, H., et al., STRONG for Surgery & Strong for Life — against all odds: intensive prehabilitation including smoking, nutrition, alcohol and physical activity for risk reduction in cancer surgery — a protocol for an RCT with nested interview study (STRONG-Cancer). Trials, 2022. 23(1): p. 1-13. | 1 |  |  |  | 1 |  |
| Trang, K. and D.A. Spain, Smoking cessation in elective surgery. American Surgeon, 2019. 85(4): p. E193-E194. | 1 |  |  |  |  |  |
| Turan, A., et al., Transdermal nicotine patch failed to improve postoperative pain management. Anesthesia and Analgesia, 2008. 107(3): p. 1011-1017. |  | 1 |  |  |  |  |
| van Rooijen, S.J., et al., Making Patients Fit for Surgery: Introducing a Four Pillar Multimodal Prehabilitation Program in Colorectal Cancer. American journal of physical medicine & rehabilitation, 2019. 98(10): p. 888-896. |  | 1 |  |  |  |  |
| Vial, R.J., et al., Smoking cessation program using nicotine patches linking hospital to the community. Journal of Pharmacy Practice and Research, 2002. 32(1): p. 57-62. |  |  | 1 |  |  |  |
| Wallstrom, M., F. Nilsson, and J.M. Hirsch, A randomized, double-blind, placebo-controlled clinical evaluation of a nicotine sublingual tablet in smoking cessation. Addiction, 2000. 95(8): p. 1161-1171. |  | 1 |  |  |  |  |
| Warner, D.O., Helping surgical patients quit smoking: Time to bring it home. Anesthesia and Analgesia, 2015. 120(3): p. 510-512. | 1 |  |  |  |  |  |
| Warner, D.O. and Y. Shi, Is it dangerous to quit smoking shortly before surgery? Anesthesiology, 2011. 115(5): p. 1137-1138. | 1 |  |  |  |  |  |
| Warner, M.A., M.B. Divertie, and J.H. Tinker, Preoperative cessation of smoking and pulmonary complications in coronary artery bypass patients. Anesthesiology, 1984. 60(4): p. 380-383. | 1 |  |  |  |  |  |
| Weaver, K.E., et al., Preliminary efficacy of an enhanced quitline smoking cessation intervention for cancer patients. Journal of Clinical Oncology, 2015. 33(15). |  | 1 | 1 |  |  |  |
| Wheatley, I.C., K.J. Hardy, and C.E. Barter, An evaluation of preoperative methods of preventing postoperative pulmonary complications. Anaesthesia and Intensive Care, 1977. 5(1): p. 56-59. |  | 1 |  |  |  |  |
| Wolfenden, L., et al., Smoking and surgery: An opportunity for health improvement [3]. Australian and New Zealand Journal of Public Health, 2007. 31(4): p. 386-387. | 1 |  |  |  |  |  |
| Yan, G., L. Wei, and Y. Shui, Effect of nurse-led peer education program on self-management behavior for coronary stent implantation patients. Journal of the American College of Cardiology, 2014. 64(16): p. C221. |  | 1 |  |  |  |  |
| Yang, G.P. and M.T. Longaker, Abstinence from Smoking Reduces Incisional Wound Infection: A Randomized, Controlled Trial. Annals of Surgery, 2003. 238(1): p. 6-8. | 1 |  |  |  |  |  |
| Young-Wolff, K.C., R. Fogelberg, and P.G. Preston, Implementing a Multifaceted Perioperative Smoking Cessation Intervention in a Large Healthcare System. Nicotine & tobacco research : official journal of the Society for Research on Nicotine and Tobacco, 2020. 22(3): p. 452-453. | 1 |  |  |  |  |  |
| Abstracts. Obstetrics & Gynecology, 2006. 108(5): p. 1293-1296. | 1 |  |  |  |  |  |
| Smoking cessation interventions and strategies. Best Practice, 2008. 12(8): p. 1-4. | 1 |  |  |  |  |  |
| CABG surgery: smoking cessation reduces mortality. Nursing, 2008. 38(12): p. 26-27. | 1 |  |  |  |  |  |
| RC currents. Surgical savings. AARC Times, 2009. 33(6): p. 77-77. | 1 |  |  |  |  |  |
| Nursing Care Partnership program: project summaries. Canadian Nurse, 2010. 106(7): p. 14-16. | 1 |  |  |  |  |  |

Footnote *incomplete data: reported 1 person quit smoking, unclear group allocation, authors contacted but no response

**Table S2: Pilot and subgroup studies (“subgroup study”: in which smokers made up only a proportion of the study population)**

| **Study Type** | **Study ID** | **Details** |
| --- | --- | --- |
| **Pilot studies** | **Myles [49]** | “Randomised, double blind, sham-controlled trial”. We have categorised this as a pilot study as the author concludes: "Statistical power analysis suggests a sample size of approximately 200 patients would be required to demonstrate a difference in smoking cessation rates, if in fact it exists." 52 patients  The pilot study underlying Myles 1996 (included in review) |
|  | **Stanislaw [59]** | total 26 patients randomised |
|  | **Wewers [66]** | total 80 patients randomised |
|  | **Warner [61]** | total 46 patients randomised |
|  | **Warner [63]** | total 130 patients randomised |
|  | **Lee [24]** | total 30 patients randomised  “In this pilot study, we sought to determine the feasibility and acceptability of e-cigarettes, compared to nicotine patch, for perioperative smoking cessation in veterans.”  Veterans hospital  Also published as a conference Abstract Lee 2017 |
|  | **Krebs [44]** | total 42 patients randomised  Outcomes not defined as primary or secondary  Specifically states that abstinence between groups was not compared as the study was not powered to show differences |
|  | **Webb [65]** | total 600 patients randomised  The pilot study underlying Webb 2022 (included in review) |
|  | **Rojewski [55]** | total 40 patients randomised  Multi-site  Comparison is not usual care vs intervention |
| **Subgroup studies** | **Allen [39]** | Women undergoing coronary artery bypass graft surgery |
|  | **McHugh [47]** | Men undergoing coronary artery bypass graft surgery |
|  | **Kadda [17]** | Patients undergoing open heart surgery |
|  | **Lauridsen [22]** | Patients who smoke tobacco, drink alcohol, or smoke tobacco and drink alcohol undergoing radical cystectomy |

**Table S3 : Full, detailed description of interventions and outcomes for each included study**

| **Intervention Timepoint** | **Study ID** | **Year and country of publication** | **Detailed Intervention** | **Detailed Outcomes** |
| --- | --- | --- | --- | --- |
| **Pre-operative**  **only** | McHugh [47] | 2001  United Kingdom | Monthly motivational interviews in the patient's own home, and record cards to track progress  Cointerventions targeting other cardiac risk factors | On admission for surgery (median of 8 months from intervention): Smoking status (self-report), obesity, physical activity, anxiety and depression, general health status, and proportion of patients exceeding target values for blood pressure, plasma cholesterol, and alcohol intake. |
|  | Myles [51] | 2004  Australia | 7 week prescription of bupropion (delivered as 3 + 4 weeks supply) plus brief advice, booklet and 1x telephone call around quit date | Daily cigarette consumption at hospital admission.  Smoking cessation (+/-28 days) on admission for surgery, at 3 weeks, 6 weeks, and 6 months post op, self-reported daily cigarette consumption at 3 and 6 weeks, pulse oximetry and expired CO concentration at hospital admission, peri-operative respiratory complications and postoperative wound infection, and duration of stay in the recovery room and in hospital |
|  | Andrews [40] | 2006  United Kingdom | **Intervention:** Letter from consultant surgeon and details of local stop smoking service (not an actual referral)  **Control:** given booklet, nurse advice | Abstinence on day of surgery (self-reported via questionnaire), intention to quit permanently (self-reported from subgroup of those who reported abstinence) |
|  | Sorensen [58] | 2007  Denmark | **Intervention:**  Pooled analysis of 2 intervention groups:  1. telephone (brief advice in clinic then 10 min telephone call approx 1 month before surgery)  2. outpatient (brief advice in clinic then 20 min face-to-face meeting in clinic, plus personalised NRT dosage advice with samples of preferred NRT method given, and advice to take until 24h before surgery)  **Control:** standard advice | Abstinence on day of surgery (“stopped smoking before operation”, assessed on day of surgery)  Abstinence day of suture removal and 3 months postoperatively  Post operative wound infection |
|  | Thomsen [60] | 2010  Denmark | **Intervention:** 1 x 45-90 min face-to-face counselling session 3-7 days preoperatively, and NRT provision    **Control:** routine preoperative information (inconsistent or no advice) | Postoperative complications  Smoking cessation from two days before to ten days after surgery, and long-term continuous cessation from two days preoperatively to twelve months postoperatively |
|  | Warner [61] | 2012  United States | **Intervention:** Nicotine lozenge  **Control: P**lacebo lozenge | self-reported morning abstinence on day of surgery  self-reported abstinence postop day 8, post op CO, time to last cigarette, preop nicotine withdrawal score |
|  | Shi [56] | 2013  United States | Intervention: Brief advice and being specifically advised about carbon monoxide testing  Control: Brief advice only | CO levels on the morning of surgery  Self-reported smoking on the day of surgery, self-reported time since last cigarette, intent to maintain abstinence on the morning of surgery |
|  | Warner [63] | 2015  United States | **Intervention**: Decision aid used in preoperative assessment clinic  **Control:** Usual care | Decisional quality and patient involvement in decision making  smoking behaviour in the perioperative period was a secondary outcome: self-report of abstinence on morning of surgery, and 30 day 7 day PPA by carbon monoxide |
|  | Bohlin [21] | 2020  Sweden | **Four intervention groups:**  **Group 1** (control, usual care)  **Group 2** (written information provided via registry)  **Group 3** (Doctor informed of smoking status)  **Group 4** (written information and doctor informed) | Rate of smoking cessation before and after surgery (6 weeks, 3-6 weeks, 1-3 weeks before surgery, week of surgery, 1-3 weeks and 3-6 weeks after surgery)  Post-operative complications (major, minor, combined surgical site and urinary tract infections) |
|  | Webb [65] | 2020  Australia | **Intervention:** literature offering access to 5 weeks free NRT patches, plus hospital's standard "quit pack"  **Control:** usual care (hospitals standard "quit pack" only) | Primary outcome was smoking abstinence for ≥ 4 weeks, as self-reported by participants on the day of surgery, including, where possible, corroboration using exhaled carbon monoxide testing  Secondary outcomes were: cessation for 24 or more hours before surgery; total quitting activity, defined as successful quits plus cessation attempts lasting for more than 24 h that ended in relapse; the use of any stop-smoking medication; cessation for at least 24 h before surgery; and self-reported smoking reduction. |
|  | Rojewski [55] | 2021  United States | **Intervention** ("CM"): standard care (counselling + nicotine replacement therapy [NRT]) + escalating schedule of monetary payment delivered contingent on abstinence confirmed by CO breath test    **Control** ("MO"): standard care (counselling + NRT) + breath tests with no payments | 7-day point prevalence abstinence (PPA) on day of surgery  7-day PPA at 3 months follow up  Intervention engagement |
| **Pre- and post-operative** | Møller [48] | 2002  Denmark | **Intervention:** weekly counselling and personalised NRT schedule, from 6–8 weeks before operation to 10 days postoperation.    **Control:** standard care "little or no information about the risk of tobacco smoking, nor any smoking cessation counselling. " | Postoperative complications: cardiopulmonary, renal, neurological, or surgical complications  Duration of hospital admittance  Moller 2002 reports abstinence at time of surgery although not a specified outcome  Abstinence was the primary outcome of the subsequent papers (30 days and 1 year) |
|  | Ratner [53] | 2004  Canada | **Intervention:**  3 components:  1. the PAC intervention (15 min face to face counselling session) plus informed of quitline  2. the in-hospital intervention (face to face counselling) plus self help cards  3. the telephone counselling intervention (weekly for the first month, biweekly for the second and third month), 9th and final support call 16 weeks post discharge    **Control:** standard care | Abstinence from smoking 24h prior to surgery ("fasting")  Abstinence at 6 and 12 months postoperatively ("cessation") |
|  | Wolfenden [67] | 2005  Australia | **Intervention:** Stratified into dependent and non-dependent smokers: all received approx 17 minute tailored computer counselling session, tailored self-help material, material to prompt brief advice by healthcare staff, and telephone counselling (2 sessions, 1x after clinic and 1x before admission for surgery); plus 1-2 weeks +/- in hospital prescription of NRT if defined as "nicotine-dependent"    **Control:** Usual care (advice at discretion of clinic staff) | Self-reported pre-operative abstinence (> 24 h before admission) and 3-month point prevalence abstinence (not smoking at the time of follow-up assessment)  Mean score on the Heaviness of Smoking Index at follow-up, the proportion of participants who had improved their stage of change between measures, annual cost of delivering intervention. |
|  | Lindström [46] | 2008  Sweden | **Intervention:** Weekly in person or telephone counselling for 4 weeks preop and 4 weeks post op, helpline number and NRT. Mean of 7 meetings per patient.    **Control:** usual care with "general advice to stop smoking" | Complications (any) at 30 days, wound complications at 30 days, short term smoking abstinence (self-reported 3 week prior to surgery and CO 2-3 weeks post operatively)    Long term smoking abstinence at 12 months by self-report using questionnaire. |
|  | Warner [62] | 2011  United States | **Intervention:**  3 components:  1. brief advice  2. information on quitline services  3. brochure including the quitline telephone number and the option of a faxed quitline referral  **Control:** Brief (5 minute) comparison intervention based on the 5As | Quitline use  Point prevalence and continuous abstinence at 30 and 90 days postoperatively |
|  | Wong [68] | 2012  Canada | **Intervention:** 2 x 15 minute standardised counselling sessions (preadmission and predischarge) and 12 weeks of varenicline    **Control:** 2x 15 min standardised counselling sessions (preadmission and predischarge) and 12 weeks of placebo tablets | 7-day point prevalence (PP) abstinence rate at 12 months  Abstinence on the target quit day (24h preop), 7-day PP abstinence at 3 and 6 months, self-reported changes in the number of cigarettes per day and stage of change at 3, 6, and 12 months, perioperative complications (any, wound, cardiopulmonary) and adverse events. |
|  | Lee [45] | 2013  Canada | **Intervention:** brief preoperative counselling (<5 mins), written resources, QuitLine referral and 6-week supply of transdermal nicotine replacement therapy    **Control:** Usual care | Rate of smoking cessation on the day of surgery (self-report 7 days, biochemically confirmed 24 hours) by exhaled carbon monoxide breath test.  Perioperative complications and unanticipated hospital admission, smoking cessation by self-report at 30 days, smoking cessation by self-report at 12 months, difference between self-report and biochemical validation, cigarettes per day, exhaled carbon monoxide, duration of surgery, length of hospital stay |
|  | Ostroff [16] | 2014  United States | **Intervention:** Best practice plus scheduled reduced smoking  (Brief intervention in preop assessment, NRT, up to 5 counselling sessions plus scheduled reduced smoking (SRS) via a handheld device called "QuitPal")    **Control:** Best practice only | Short (hospital admission and three months) and longer-term (6 months) biochemically verified smoking abstinence.  Treatment implementation, use and fidelity |
|  | Kehlet [43] | 2015  Denmark | **Intervention:** The Gold Standard Programme and the offer of free NRT    **Control:** "the hospital department's standard of care" and the offer of free NRT | Perioperative complications at 30 days  Abstinence at 6 weeks |
|  | Wong [20] | 2017  Canada | **Intervention:** (1) 10- to 15-minute structured preoperative counselling session; (2) pharmacotherapy with a free 3-month supply of varenicline; (3) an educational pamphlet; and (4) a fax referral to a quitline for proactive telephone counselling and follow-up    **Control:** brief advice regarding smoking cessation (3-5 mins) and quitline information for self-referral. | 7-day point prevalence (PP) abstinence at 12 months after surgery  PP and continuous abstinence at 1, 3, and 6 months, change in the number of cigarettes per day, Fagerström test score, and the Stage of Change at 1, 3, 6, and 12 months, quitline use, incidence of perioperative complications as well as medication-related adverse events  Multivariable generalized linear regression was used to identify independent variables related to abstinence. |
|  | Lee [24] | 2018  United States | **Two intervention groups:**  **Nicotine patch group** - 6 weeks supply, tapering dose  **E cigarette group** - 6 weeks supply, tapering dose    **Control:** All patients received brief counselling, brochure and Quitline referral | Primary outcome: rate of smoking cessation on day of surgery (CO breath test confirmed)  Secondary outcomes: included smoking habits and pulmonary function on the day of surgery and at 8 weeks |
|  | Lauridsen [22] | 2022  Denmark | **Intervention:** Gold Standard Programme plus NRT, chlordiazepoxide and disulfiram if appropriate. Multimodal intervention targeting smoking and alcohol.  **Control:** usual care | Prevalence of any postoperative complication, or death, within 30 d after surgery  Abstinence (from smoking and/or alcohol) at 30 days and 12 months, health-related quality of life, length of stay, time back to habitual activity, and mortality. |
|  | Webb [23] | 2022  Australia | **Intervention:** Brochure, printed offer of NRT and quitline support, 1x telephone meeting to discuss support and provide dosed NRT/onward quitline support    **Control:** standard care (a brochure on smoking and surgery | Quitting at least 24 hours before surgery, (where possible: see note) verified by exhaled carbon monoxide testing  Quitting at least four weeks before surgery, adverse events, and (for those who had quit before surgery) abstinence three months after surgery. |
| **Intra-operative**  **only** | Myles [49] | 1992  Australia | **Intervention:** An audiotape encouraging smokers to give up smoking  **Control:** sham audiotape recording | Desire to quit smoking 1 day postoperatively  (Visual Analogue Scale)  Abstinence at 1 month and 6 months  Change in smoking behaviour at 1 month and 6 months |
|  | Hughes [42] | 1994  United Kingdom | **Intervention:** Delivery of recorded audiotaped message on a loop while under general anaesthesia    **Control:** Delivery of sham message (matched for number of syllables) on a loop while under general anaesthesia | Smoking habits one month post operatively (reduced, stopped or no change and increased)  Daily cigarette consumption |
|  | Myles [50] | 1996  Australia | **Intervention:** Delivery of recorded audiotaped message on a loop while under general anaesthesia    **Control**: Delivery of sham message (blank tape) on a loop while under general anaesthesia | Abstinence at 2 months (self-report) and 6 months (biochemically validated) |
| **Post-operative**  **only** | Rigotti [54] | 1994  United States | **Intervention:** Three session program, video and face to face counselling, counselling time 60 mins, plus telephone call 1 week post discharge  **Control:** usual care "including brief advice not to smoke" | Smoking status at 1 year and 5.5 years postoperatively  Smoking status at hospital discharge and 2 weeks and 2, 4, 8, and 10 months later. |
|  | Stanislaw [59] | 1994  United States | **Intervention:** Structured postoperative smoking cessation intervention (3 visits, literature and a relaxation audiotape) during hospitalisation followed by five weekly phone calls after discharge  **Control:** usual care | Abstinence at first post discharge visit (5 weeks post discharge) |
|  | Wewers [66] | 1994  United States | **Intervention:** Structured postoperative smoking cessation intervention (3 visits, literature and a relaxation audiotape) during hospitalisation followed by five weekly phone calls after discharge  **Control:** usual care (patients on the CV unit only were given booklet that discusses risk factors including smoking cessation advice) | Abstinence at first post discharge visit (5 weeks post discharge)  Abstinence rates within each group (CV, oncology, general)  Saliva cotinine levels for those who continued to smoke |
|  | Allen [39] | 1996  United States | **Intervention:** Multimodal intervention targeting cardiac risk factors: 3 postoperative in-person meetings and 1 telephone follow up (discharge, 2 weeks, 1 month, 2 months) and visual/written resources    **Control:** Usual care | Smoking cessation (change in prevalence from baseline), dietary changes, body composition, physical activity. All assessed at 12 months. |
|  | Simon [57] | 1997  United States | **Intervention:**  4 components:  1. Counselling: 1x Face-to-face session (30-60 mins) plus 10 minute videotape, then 5x (<30 minute) follow up telephone calls (weekly for a month then monthly for 2 months)  2. 3 months of NRT if no contraindication  3. psychology referral if depression suspected  4. self-help literature    **Control:** brief counselling (10 mins) before discharge and a self-help leaflet | Smoking status at 6 and 12 months  Number of quit attempts, date of last cigarette, longest period of abstinence, duration of NRT at 6 and 12 months |
|  | Griebel [41] | 1998  United States | **Intervention:** Post-operative counselling (1x 20 minute meeting, 5x 10 minute weekly phone calls with the same ANP) booklet resources  **Control:** Usual care | Smoking status (7 day abstinence) at outpatient visit 6 weeks after discharge  Change in number of cigarettes smoked per day, documented smoking cessation intervention on patient chart |
|  | Warner [64] | 2005  United States | **Intervention:** Nicotine patches  **Control:** Placebo patches | Perceived stress score  Numeric stress score, numeric pain score, nicotine withdrawal score, 7 day point prevalence abstinence at 30 days and 6 months, continuous abstinence at 30 days and 6 months |
|  | Nåsell [52] | 2010  Sweden | **Intervention:** 1-2 in person meetings and weekly telephone contact for 6 weeks, offer of NRT  **Control:** general advice to stop smoking, but no additional support | Prevalence of one or more post operative complication at 2 and 6 weeks post operatively  Abstinence from smoking at 2 and 6 weeks post operatively |
|  | Kadda [17] | 2015  Greece | **Intervention:** Multimodal intervention with counselling (2 hour in person session prior to discharge, 1 year of monthly telephone contact) Targeting lifestyle and cardiac risk factors.    **Control:** Usual care (general verbal advice from hospital staff) | The development of a non-fatal cardiovascular event at one year  Fatal events, smoking abstinence, dietary habits and a physical activity evaluation all at one year |
|  | Krebs [44] | 2019  United States | **Intervention:** "QuitIT" application (smoking cues coping skills game) plus standard care    **Control:** Standard care (tele-counselling and cessation pharmacotherapies) | Feasibility and game-use metrics  "Tobacco-related factors" at 1 month postoperatively: situational self-efficacy, desire to quit, days of abstinence post discharge |
|  | Matuszewski [6] | 2021  United States | **Two intervention groups:**  **Brief:** 10 minute counselling session prior to discharge and Quit Line referral  **Extended:** as for Brief plus repeat follow-up by counsellor at standard 2 weeks, 6 weeks, 3 months, and 6 months.  **Control:** usual care | Smoking cessation confirmed by exhaled carbon monoxide at 3 and 6 months.  Proportion accepting services from a nationally based Quitline. |

**Table S4: Studies with > 20% loss to follow up and < 80% eligible recruitment**

| **Study ID** | **% drop out** | **% eligible participants not randomised** |
| --- | --- | --- |
| **Webb [65]** | **79.17** | **NR** * |
| **Bohlin [21]** | **54.38** | **0.00** |
| **Webb [23]** | **32.28** | **-1.87** |
| **Ratner [53]** | **28.69** | **20.69** |
| **Warner [62]** | **23.00** | **37.89** |
| **Griebel [41]** | **22.22** | **41.94** |
| **Rojewski [55]** | **22.22** | **4.76** |
| Hughes [42] | 18.03 | NR |
| Allen [39] | 15.94 | 20.69 |
| Warner [64] | 15.94 | 20.69 |
| Wolfenden [67] | 15.94 | 20.69 |
| Sorensen [58] | 15.94 | 9.09 |
| Thomsen [60] | 15.94 | 20.69 |
| Wong [68] | 15.94 | 20.69 |
| Ostroff [16] | 15.94 | 29.92 |
| Kehlet [43] | 15.94 | 20.69 |
| Lee [24] | 15.94 | 87.80 |
| Krebs [44] | 15.94 | 20.69 |
| Lindstrom [46] | 12.82 | 50.84 |
| Wong [20] | 11.82 | 32.11 |
| McHugh [47] | 10.74 | 20.69 |
| Myles [50] | 10.19 | NR |
| Møller [48] | 10.00 | 27.71 |
| Lauridsen [22] | 9.62 | 20.69 |
| Warner [63] | 9.23 | 40.91 |
| Matuszewski [6] | 7.89 | 20.69 |
| Simon [57] | 7.72 | 20.69 |
| Lee [45] | 6.55 | 66.06 |
| Nåsell [52] | 3.81 | 20.69 |
| Shi [56] | 2.73 | -8.28 |
| Rigotti [54] | 2.25 | 25.83 |
| Myles [51] | 0.98 | NR |
| Andrews [40] | 0.98 | NR |
| Myles [49] | 0.00 | NR |
| Wewers [66] | 0.00 | 11.11 |
| Stanislaw [59] | 0.00 | 7.14 |
| Warner [61] | 0.00 | NR |
| Kadda [17] | 0.00 | 6.89 |

Studies are listed in decreasing order of percentage drop out rates. Loss to follow up was assessed at conclusion of the trial follow up period.

* NR = not reported. Studies in blue: trial did not report number of patients that were eligible for inclusion. Studies in bold: >20% drop-out.

**Table S5** **Characteristics of the 38 randomised controlled trials included in the systematic review: type of surgery**.

Studies have been grouped by type of surgery.

|  | **Surgery Classification Summary** | **Study ID** |
| --- | --- | --- |
| **Mixed Elective** | Elective surgery, mixed | Myles [50] |
|  | Elective surgery, mixed | Simon [57] |
|  | Elective surgery, mixed | Myles [51] |
|  | Elective surgery, mixed | Warner [64] |
|  | Elective surgery, mixed | Andrews [40] |
|  | Elective surgery, mixed | Warner [62] |
|  | Elective surgery, mixed | Warner [61] |
|  | Elective surgery, mixed | Lee [45] |
|  | Elective surgery, mixed | Shi [56] |
|  | Elective surgery, mixed | Warner [63] |
|  | Elective surgery, mixed | Lee [24] |
|  | Elective surgery, mixed; women only | Hughes [42] |
|  | Elective surgery, mixed (including cardiac) | Ratner [53] |
|  | Elective surgery, mixed (not cardiac) | Wolfenden [67] |
|  | Elective surgery, mixed (not cardiac) | Wong [68] |
|  | Elective surgery, mixed (not cardiac) | Wong [20] |
|  | Elective surgery, mixed (not cardiac, not neuro) | Webb [23] |
|  | Elective surgery, mixed (not cardiac, not neuro) | Webb [65] |
| **Cardiac** | Cardiac (CABG only) | Rigotti [54] |
|  | Cardiac (CABG only) | McHugh [47] |
|  | Cardiac, mixed (CABG and other) | Kadda [17] |
|  | Cardiac; first time CABG only; women only | Allen [39] |
| **Other specific subspecialities** | Acute trauma surgery | Nåsell [52] |
|  | Acute trauma surgery | Matuszewski [6] |
|  | Breast surgery (cancer and other) | Thomsen [60] |
|  | Gynaecological surgery (cancer and other) | Bohlin [21] |
|  | Cancer surgery, mixed | Stanislaw [59] |
|  | Cancer surgery, mixed | Griebel [41] |
|  | Cancer surgery, mixed | Ostroff [16] |
|  | Cancer surgery, mixed | Krebs [44] |
|  | Cancer surgery, mixed | Rojewski [55] |
|  | Radical cystectomy for bladder cancer | Lauridsen [22] |
|  | Cancer, cardiac and general surgery | Wewers [66] |
|  | General surgery | Myles [49] |
|  | Elective general surgery | Sorensen [58] |
|  | Elective orthopaedic and general surgery | Lindström [46] |
|  | Elective orthopaedic surgery | Møller [48] |
|  | Vascular surgery | Kehlet [43] |

**Table S6:** **Results of a sensitivity analysis excluding studies more than 20 years old**

| **Outcome** | **Meta-analysis results with all relevant studies included** | **Meta-analysis results excluding studies >20 years old** |
| --- | --- | --- |
| Abstinence at time of surgery | RR (95% CI) 1.48 (1.20-1.83)  Chi^2^ =85.89 df=20 p<0.001 | RR (95%CI) 1.33 (1.11-1.60)  Chi^2^ =59.16 df=18 p<0.0001 |
| Abstinence at 12 months post-operatively | RR (95%CI) 1.62 (1.29-2.03)  Chi^2^ =15.43 df=10 p=0.12 | RR (95%CI) 1.59 (1.28-1.98)  Chi^2^ =6.95 df=6 p=0.33 |

**Table S7:** **Published protocols for future trials which, when completed and published, will likely meet the criteria for subsequent updates to this review**

| **Protocol Name** | **Methods** | **Aim** | **Participants** | **Interventions** | **Outcomes** | **Notes** |
| --- | --- | --- | --- | --- | --- | --- |
| **Bahia 2015 [1]** | Pilot RCT | Estimating enrolment to a trial of CR after A/TAA repair    Estimating compliance with CR amongst patients with A/TAA. | Patients with abdominal and thoracic aortic aneurysms (A/TAA) after aneurysm repair at 2 sites in the UK    Aiming to randomised 84 participants | Intervention: cardiac rehabilitation    Control: Standard care | The primary outcome measures are enrolment in the RCT and compliance with CR.    Secondary outcomes will include phenotypic markers of cardiovascular risk and smoking cessation, alongside disease-specific and generic quality-of-life measures. | A multimodal intervention that included smoking cessation    Will likely include a subgroup of smokers |
| **Bendtsen 2019 [2]** | Pilot RCT | To evaluate the effectiveness of an SMS-based intervention on smoking behavior of patients undergoing elective surgery. | Smokers undergoing elective surgery in 20 surgical departments in the South East of Sweden    Aiming to randomise 434 participants | Intervention: novel 12 week SMS program (daily SMS messages with behaviour change enhancing content and access to interactive modules)    Control: usual care | Primary outcome measures, prolonged abstinence, and point prevalence of smoking cessation will be measured through questionnaires at 3, 6, and 12 months after randomization.    Logistic regression models adjusted using baseline characteristics will be explored to identify potential effects of the intervention. |  |
| **Durrand 2022 [3]** | Systematic development and feasibility testing project | We aim to systematically develop and test the feasibility of an evidence and theory-informed multi-behavioural digital prehabilitation intervention ‘iPREPWELL’ designed to prepare patients for major surgery. The intervention will be developed with reference to the Behaviour Change Wheel, COM-B model, and the Theoretical Domains Framework…The multifaceted and systematically developed intervention will be the first of its kind and will provide a foundation for further refinement prior to formal efficacy testing. | Patients preparing for major surgery and healthcare professionals 45 involved with their clinical care from two UK National Health Service centres | Stage 1 (systematic development): COM-B 48 questionnaire and to take part in a qualitative interview study and co-design workshops    Stage 2 (feasibility testing of the intervention): single group intervention study | Stage 2: primary outcomes will include feasibility, acceptability, and fidelity of intervention delivery, receipt, and enactment |  |
| **Fankhauser 2022 [4]** | Randomised, single blinded, controlled superiority trial | The primary objective is to compare complications between patients with an institutional multifaceted smoking cessation intervention starting 4 weeks before surgery compared to patients in the advice-only group (control group) within a 90-day postoperative period. | Patients listed for intermediate or high-risk surgery (abdominal, thoracic, urology, gynaecology, vascular or head and neck surgery) at a single centre    Aim to randomise 251 patients | Intervention: Interview by tobacco treatment specialist 4 weeks preoperatively with individualised counselling and nicotine replacement therapy    Control: usual care | The primary endpoint is the Comprehensive Complication Index (CCI®) within 90 days of surgery.    Secondary outcomes include the length of hospital stay, cost of care, quality of life, smoking abstinence, and reduction in nicotine consumption at 3, 6 and 12 months |  |
| **Hartog 2019 [5]** | Prospective, randomised, open, blinded endpoint (PROBE) trial | To assess whether a combined preoperative and postoperative multidisciplinary cardiac rehabilitation (CR) programme (Heart-ROCQ programme) can improve functional status and reduce surgical complications, readmissions, and major adverse cardiac events (MACE) as compared with standard care. | Patients undergoing cardiac surgery in a single centre, having been referred from 4 hospitals.    Aim to randomise 350 patients | Intervention: The Heart-ROCQ programme consists of a preoperative optimisation phase while waiting for surgery (three times per week, minimum of 3 weeks), a postoperative inpatient phase (3 weeks) and an outpatient CR phase (two times per week, 4 weeks). Patients receive multidisciplinary treatment (eg, physical therapy, dietary advice, psychological sessions and smoking cessation).    Control: Standard care consists of 6 weeks of postoperativeoutpatient CR with education and physical therapy (two times per week). | The primary outcome is a composite weighted score of functional status, surgical complications, readmissions and MACE, and is evaluated by a blinded endpoint committee. The secondary outcomes are length of stay, physical and psychological functioning, lifestyle risk factors (including smoking behaviour), and work participation. Finally, an economic evaluation is performed. Data are collected at six time points: at baseline (start of the waiting period), the day before surgery, at discharge from the hospital, and at 3, 7 and 12 months postoperatively. | A multimodal intervention that included smoking cessation    Will likely include a subgroup of smokers |
| **Lugg 2020 [6]** | Stepped wedge cluster randomised controlled trial | To determine in patients who undergo major elective thoracic surgery whether an intervention integrated (INT) into the surgical pathway improves smoking cessation rates compared with usual care (UC) of standard community/hospital-based NHS smoking support. This pilot study will evaluate feasibility of a substantive trial. | Patients who undergo major elective thoracic surgery in five adult thoracic centres in the UK    Aim to recruit 120 participants | Intervention: pharmacotherapy and a hybrid of behavioural support delivered by the trained healthcare practitioners (HCPs) in the thoracic surgical pathway and a complimentary web- based application    Control: Usual care | Primary outcome: the number of patients who agree to participate in the intervention as a proportion of those eligible to enter the study.    Secondary outcomes:  1. Integration of the intervention into the clinical pathway by time from decision to operate from study recruitment.  2. Barriers to study recruitment  3. Fine- tune study procedures and pilot data capture forms  4. The proportion of patients in the intervention group who have quit smoking by the day of surgery and 1 month after surgery.  5. To assess the proportion of patients in the observation only UC group who have quit smoking by the day of surgery and 1 month after surgery.  6. To define the variability of smoking cessation practices in all patients using the nicotine replacement usage questionnaire.  7. Qualitative interview: to understand patients’ experiences of and engagement with the intervention, and any unintended consequences; to establish whether the intervention is acceptable to thoracic surgery patients and staff and investigate recommendations for optimisation of intervention delivery. |  |
| **Rojewski 2021 [7]** | Randomised controlled trial | To evaluate the effectiveness of financial incentives delivered contingent on biochemically verified smoking abstinence (contingency management (CM)) in patients with cancer undergoing surgery. | Patients who smoke, are diagnosed with or suspected to have any type of operable cancer and have a surgical procedure scheduled in the next 10 days to 5 weeks in one of two study site    Aim to randomised 282 participants | Intervention ("Contingency Management"): standard care (counselling + nicotine replacement therapy [NRT]) + escalating schedule of monetary payment delivered contingent on abstinence confirmed by CO breath test    Control ("Monitoring Only"): standard care (counselling + NRT) + breath tests with no payments | Point prevalence abstinence (PPA) outcomes (self-report of 7-day abstinence confirmed by CO≤4 ppm and/or anabasine ≤2 ng/mL) will be assessed on the day of surgery and 6 months after surgery.    The effect of CM on 7-day PPA at the time of surgery and 6-month follow-up will be modelled using generalised linear mixed effects models. | Pilot study used in the development of this protocol included in this review |
| **Tonnesen 2022 [8]** | Randomised controlled trial with nested interview study | To compare the efficacy of the integrated STRONG programme with standard care on preoperative risk reduction and secondly on SNAP factor improvement and frailty, postoperative complications, and quality of life | Patients undergoing adjuvant chemotherapy prior to radical bladder cancer surgery, who have ≥1 "risky lifestyle" (Smoking, malNutrition, obesity, risky Alcohol intake and insufficient Physical activity [SNAP]) factor.    Aim to randomise 42 patients | Intervention: Individually tailored "STRONG" programme (≥6 weekly sessions with patient education, motivational and pharmaceutical support)    Control: Usual care | Surgical risk reduction (≥1 step for 1 or more risky lifestyles on the ASA-score, secondly as having no risky SNAP factors, and as any SNAP improvement)    Postoperative complications    At surgery, 6 weeks, and 6 months postoperatively | A multimodal intervention that included smoking cessation    Will likely include a subgroup of smokers |

**References**

1. Bahia SS, Holt P, Ray KK et al. Cardiac rehabilitation versus standard care after aortic aneurysm repair (Aneurysm CaRe): study protocol for a randomised controlled trial. *Trials*. 2015; **16**(1).
2. Bendtsen M, Linderoth C, Bendtsen P. Mobile Phone-Based Smoking-Cessation Intervention for Patients Undergoing Elective Surgery : Protocol for a Randomized Controlled Trial. *JMIR research protocols* 2019; **8**(3): e12511-NA.
3. Durrand J, Livingston R, Tew G, et al. Systematic development and feasibility testing of a multibehavioural digital prehabilitation intervention for patients approaching major surgery (iPREPWELL): A study protocol. PLoS ONE. 2022; **17**(12): e0277143
4. Fankhauser CD, Affentranger A, Cortonesi B, et al. Preoperative smoking cessation program in patients undergoing intermediate to high-risk surgery: a randomized, single-blinded, controlled, superiority trial. *Trials* 2022; **23**(1): 717.
5. Hartog J, Blokzijl F, Dijkstra S, et al. Heart Rehabilitation in patients awaiting Open heart surgery targeting to prevent Complications and to improve Quality of life (Heart-ROCQ): study protocol for a prospective, randomised, open, blinded endpoint (PROBE) trial. *BMJ Open* 2019; **9**(9): e031738.
6. Lugg ST, Kerr A, Kadiri S, et al. Protocol for a feasibility study of smoking cessation in the surgical pathway before major lung surgery: Project MURRAY. *BMJ Open* 2020; **10**(11): e036568
7. Rojewski AM, Fucito LM, Baker NL, et al. Preoperative contingency management intervention for smoking abstinence in cancer patients: trial protocol for a multisite randomised controlled trial. *BMJ Open* 2021; **11**(6): e051226
8. Tønnesen H, Lydom LN, Joensen UN, Egerod I, Pappot H, Lauridsen SV. STRONG for Surgery & Strong for Life - against all odds: intensive prehabilitation including smoking, nutrition, alcohol and physical activity for risk reduction in cancer surgery - a protocol for an RCT with nested interview study (STRONG-Cancer). *Trials* 2022 **23**(1): 333
